# Exploring Moroccan Medicinal Plants for Anticancer Therapy Development Through In Silico Studies

**DOI:** 10.3390/ph17111528

**Published:** 2024-11-13

**Authors:** Amal Bouribab, El Mehdi Karim, Meriem Khedraoui, Oussama Abchir, Abdelkbir Errougui, Yasir S. Raouf, Abdelouahid Samadi, Samir Chtita

**Affiliations:** 1 Laboratory of Analytical and Molecular Chemistry, Faculty of Sciences Ben M’Sik, Hassan II University of Casablanca, Casablanca 20600, Morocco; bouribabamal@gmail.com (A.B.); 2013karim.mehdi@gmail.com (E.M.K.); meriemkhedraoui5@gmail.com (M.K.); oussamaabchir12@gmail.com (O.A.); a_errougui@yahoo.fr (A.E.); 2 Department of Chemistry, College of Science, United Arab Emirates University, Al Ain P.O. Box 15551, United Arab Emirates; yasir.raouf@uaeu.ac.ae

**Keywords:** angiogenesis, cancer, EGFR, VEGFR2, Moroccan medicinal plants, virtual screening, ADMET, molecular dynamics, *Ajuga iva* L.

## Abstract

Background: Angiogenesis is a crucial process in the growth and proliferation of cancer, enabling tumor growth through the formation of new vasculature and the supply of nutrients and oxygen to growing malignant cells. This disease-promoting process can be targeted through the inhibition of tyrosine kinase enzymes. Objectives: The objective of this study is to evaluate the anticancer potential of various Moroccan plants from different regions. While these plants have a rich history of traditional medicinal use, they have not been extensively investigated as anticancer therapies. Methods: This study employed a multifaceted approach to evaluate the anticancer potential of various Moroccan plants. Receptor–ligand docking and virtual screening were used to assess the binding affinity of phytocompounds to the EGFR and VEGFR2 receptors. Additionally, predictive pharmacokinetic analyses were conducted to evaluate the ADMET properties of the selected compounds, followed by molecular dynamics simulations to analyze the stability of the receptor–ligand complexes. Results: In our research, we identified three notable active compounds—catechin, 4-*O*-glucoside ferulic acid, and 3-glucoside resveratrol—in the Moroccan plant *Ajuga iva* L. These findings suggest that *Ajuga iva* L. may possess significant potential for cancer inhibition. Conclusions: This research highlights the potential of the Moroccan plant *Ajuga iva* L. as a source of active compounds with significant anticancer properties. Further investigation is essential to validate these findings and explore new therapeutic avenues based on these traditional resources.

## 1. Introduction

Since its initial observation, cancer has always been a major health issue, with a remarkably high incidence [1]. To this day, cancer remains one of the leading causes of death worldwide despite extensive scientific advances and ever-growing treatment options in the last 50 years [2]. Abnormal vasculature is one of the most common hallmarks of a growing tumor, and it is generally a result of dysregulated angiogenic proteins (e.g., VEGF, bFGF, IL-8, PDGF, MMPs, endoglin, tissue factor, and hypoxia tissue factor). These important biomarkers also affect the activity of the immune cells disrupting migration and activation within the tumor microenvironment. As such, the molecular inhibition of angiogenesis has been widely studied as a tractable strategy to inhibit (and ultimately halt) tumorigenesis through the blockade of nutrients and the oxygen supply [3]. Two important pro-angiogenic factors in the cell include vascular endothelial and epidermal growth factor receptors (VEGFR and EGFR, respectively) [4,5]. Both of these receptor tyrosine kinases (RTKs) consist of several human isoforms (e.g., VEGFR1–3 and EGFR1–4). Each receptor serves as an essential regulator of cellular activity and is involved in several important processes, including the proliferation, differentiation, migration, and apoptosis of tumor cells. Additionally, they impact the mechanisms related to cellular survival and interactions within the tumor microenvironment [6,7]. It is widely established that VEGFR-2 is commonly upregulated in most solid cancers, including ovarian [8], breast [9], lung [10], colorectal [11], and gastric tumors [12], while EGFR is overexpressed in triple-negative breast cancer (TNBC) [13], colon, head-and-neck, renal, ovarian, and non-small-cell lung cancer [14]. By inhibiting EGFR, it is possible not only to reduce VEGF expression but also to disrupt the growth and survival mechanisms of tumor cells, which contributes to decreasing angiogenesis [15]. However, this inhibition can lead to an upregulation of VEGFR-2, thereby increasing the risk of resistance to treatments targeting EGFR [16]. Thus, the strategy of simultaneously targeting EGFR and VEGFR-2 appears to be promising in the fight against cancer, acting synergistically to curb tumor growth and limit the formation of new blood vessels [17]. Furthermore, both receptors possess an ATP binding site, allowing various small molecules to function as dual inhibitors, such as gefitinib, erlotinib, and sorafenib [18]. As for targeted inhibition strategies, many plant-derived phytochemicals (e.g., polyphenols, terpenoids, glycosides, and alkaloids) have been known to hold medicinal utility potential as potential anticancer therapies. If optimized correctly, these compounds can be used to induce apoptosis, inhibit tumor growth, suppress angiogenesis/metastasis, cause cell cycle arrest (G2/M phase), activate caspases, and enhance human immune responses [19,20]. Traditionally, approximately 31.16% of cancer patients in Morocco rely on local medicinal plants to alleviate disease symptoms. In this regard, the Moroccan flora are among the richest and most diverse in the world, featuring nearly 4200 species, with 22% of these being endemic to the country [19]. If we consider the history of cancer drug discovery, phytocompounds have been extensively studied in the design and development of novel oncology therapies [21,22]. At the same time, tremendous advances have occurred in the past 25 years regarding the use of computational approaches such as virtual screening (VS), molecular dynamics (MD), and predictive Absorption, Distribution, Metabolism, Excretion, and Toxicity (ADMET) algorithms to enable structure-guided drug design. The use of software to expedite drug discovery has gained global adoption due to its efficiency, relative speed, cost-effectiveness, and predictive capacities. However, the computational methods also present limitations stemming from the complexity of utilizing software on clusters and the high computational power required for simulations, which can restrict the number of compounds that can be analyzed [23,24]. Computational phytochemistry, a product of the digital age, uses mathematical algorithms, statistics, and large databases to integrate theories and modeling of phytocompounds with known experimental observations as potential medicinal agents [25]. In fact, this approach has been used to assess various biological activities beyond oncology, including its applications in terms of anticoagulants [26], antioxidants [27], anti-Alzheimer therapies [28], and antidiabetic molecules [29]. Our study aims to use a series of computational experiments to explore the anticancer activity of 386 small molecules from 22 phytochemical scaffolds derived from six different plant families collected from various regions of Morocco. These include: *Amaranthaceae*, *Asteraceae*, *Fabaceae*, *Lamiaceae*, *Pinaceae,* and *Apiaceae*.

## 2. Results and Discussion

### 2.1. Molecular Docking

Molecular docking was conducted for the entire ligand library, including two positive controls, against the two receptors, EGFR and VEGFR2, starting with high-throughput virtual screening (HTVS). Following this, Glide SP and then Glide XP were used to identify the most active compounds. For VEGFR2, the docking score ranges of the ligands spanned values from −15.2 kcal/mol to −9.7 kcal/mol, while, for EGFR, they ranged from −15.3 kcal/mol to −9.3 kcal/mol. These results indicate that 27 compounds from the initial 386 compound library had a higher affinity than the reference drug in both receptors EGFR (−9.2 kcal/mol) and VEGFR2 (−9.7 kcal/mol), as shown in Table 1. Further, 19 of these 27 hits were also found to be multi-target ligands with good docking scores against both VEGFR and EGFR proteins.

### 2.2. ADMET

To evaluate the safety and pharmacokinetic properties of the compounds, an ADMET study was initially conducted to determine whether these compounds adhered to Lipinski’s rules. As shown in Table 2, the tested natural products were complied with Lipinski’s criteria, which include a molecular weight below 500 g/mol, a logP under 5, no more than ten hydrogen bond acceptors, and no more than five hydrogen bond donors. These criteria indicate their potential to be good candidates for oral drugs. To explore this, we assessed their pharmacokinetic properties and toxicity. As shown in Table 3, all the compounds were soluble in water, with logP values ranging from −3.78 to −1.99. Among the phytocompounds examined, compound **296** displayed high predictive permeability through Caco-2 cells, indicating strong intestinal absorption and potentially high oral bioavailability. In contrast, the other compounds exhibited no permeability through Caco-2 cells, with predictive values below 0.9. Regarding intestinal absorption, most molecules were permeable, except for compounds **4**, **100**, **106**, **286**, **298**, and **331**, which had absorption rates below 30%. None of the tested compounds were able to cross the blood–brain barrier. In terms of metabolism, ligands **7**, **22**, and **282** inhibited the detoxification enzyme cytochrome P450 CYP1A2, while compounds **276** and **296** inhibited the CYP1A2, CYP2C19, and CYP2C9 isoforms. In terms of excretion, the predicted total clearance values ranged from 0.12 to 0.71 log mL/min/kg, with compound **326** showing the lowest value and compound **296** the highest. Toxicity assessments revealed that compound **298** exhibited hepatotoxicity, while compound **282** tested positive for mutagenicity in the AMES test. Furthermore, ligand **326** was found to potentially inhibit the hERG II potassium channel, indicating a possible risk for cardiovascular complications. Due to their toxicity, these compounds were eliminated from the study. In contrast, the other compounds demonstrated safety, with a maximum tolerated dose ranging from 0.13 to 1.05 log mg/kg/day, as shown in Table 4. For the lethal dose (LD50), the safe compounds showed rather similar values, ranging from 1.82 to 3.70 mol/kg. The LOAEL test identified values between 2.01 and 5.12 log mg/kg_bw/day.

### 2.3. Molecular Dynamics

Based on virtual docking screening and ADMET analysis. three molecules showed promising results at the EGFR and VEGFR2 receptors, as shown in Table 5. In this study, we simulated the interaction of these three ligands with the two receptors and compared them with reference molecules. Their stability and conformational changes were assessed during a 100 ns simulation by calculating the parameters RMSD, RMSF, Rg, SASA, and protein–ligand contacts.

#### 2.3.1. Root Mean Square Deviation

**Figure 1:**
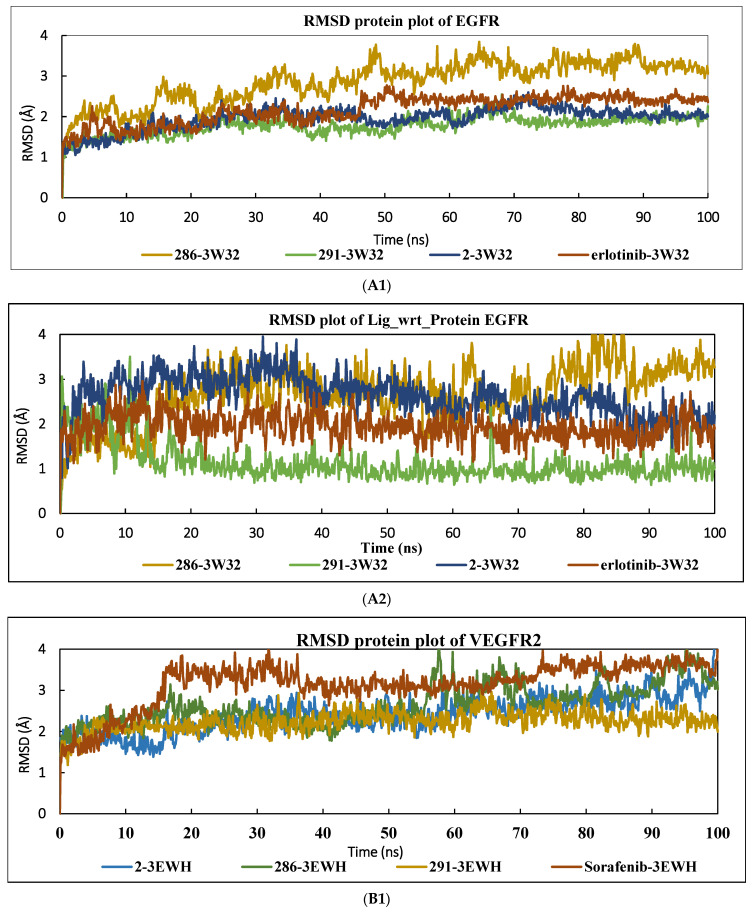

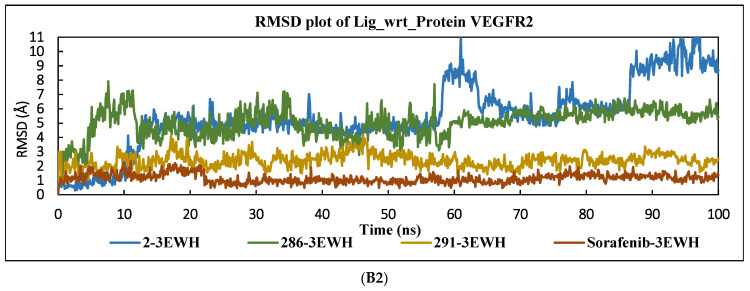
RMSD plots of proteins and ligands for the complexes 2-EGFR, 286-EGFR, 291-EGFR, and Erlotinib-EGFR (**A1**,**A2**), as well as for 2-VEGFR2, 286-VEGFR2, 291-VEGFR2, and Sorafenib-VEGFR2 (**B1**,**B2**).

The RMSD of the protein and ligand was calculated for each complex to monitor their stability and movement over 100 ns. As shown in Figure 1, the RMSD for the EGFR protein was stable after 50 ns, with small fluctuations and values ranging from 1 to 3.7 Å. However, the RMSD curve for the ligand in the EGFR protein complex (291-3W32) showed destabilization during the first 20 ns, with RMSD values varying between 1 and 2.4 Å before becoming stable. The relative RMSD values for the ligand in the 286-3W32 and 2-3W32 complexes ranging from 1 to 4 Å were found to be higher than those for the drug-bound reference protein, which ranged from 1 to 3 Å. In contrast, the 291-3W32 system showed lower RMSD values than the reference drug, oscillating between 0.5 and 2 Å but with fluctuations of 3.5 Å at the beginning of the first 30 ns. Thereafter, stabilization was observed for the rest of the simulation. For the VEGFR2 protein plot, all the complexes showed lower RMSD values than the reference drug, as indicated in Figure 1. For the RMSD plot for the reference drug, the ligand showed RMSD values varying between 1 and 2 Å, with small fluctuations at the beginning of the simulation. In comparison, the studied ligands bound to the 3W32 protein showed higher RMSD values than Sorafenib, ranging between 1 and 12 Å due to fluctuations observed during the first 60 ns of the simulation, with the exception of the 2-3EWH complex, which showed large fluctuations throughout the trajectory. These variations and fluctuations in the RMSD values indicate significant conformational changes in the protein’s active sites. The initial fluctuations of the ligands studied suggest a period of structural adjustment before stabilizing their interaction with the protein. In particular, the 2-3EWH complex, with its large fluctuations, could indicate a less stable interaction or a more dynamic conformational adjustment than the other complexes.

#### 2.3.2. Root Mean Square Fluctuations

**Figure 2:**
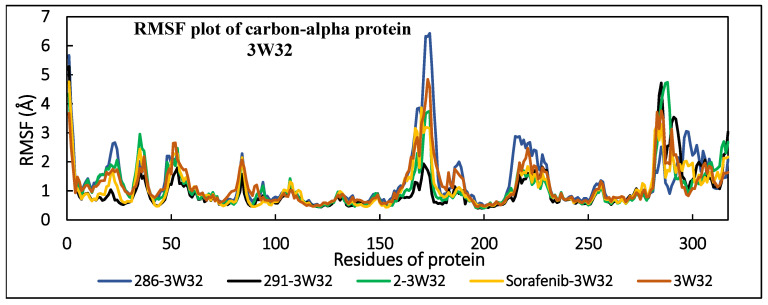

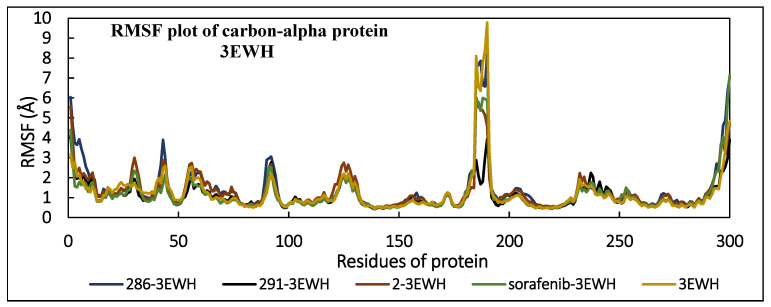
RMSF of Cα atoms of EGFR and VEGFR2 in the absence and presence of the investigated ligands (**2**, **286**, **291**, and reference drug).

The RMSF of the alpha carbon (Cα) atoms for all the residues in both proteins (VEGFR2 and EGFR) was calculated during the simulation to assess the dynamic behavior of these residues. The RMSF analysis offers important insights into the structural flexibility and fluctuations across various regions of the proteins. Generally, higher RMSF values suggest instability of the residues, while lower values indicate greater stability. The RMSF of the Ca atoms of the complexed and uncomplexed 3W32 protein are shown in Figure 2. The majority of the residues exhibited RMSF values of no more than 3 Å, indicating their stability. However, in the 286-3W32 complex, some residues displayed significant fluctuations, with RMSF values exceeding 6 Å, surpassing those of the non-complexed protein and indicating increased movement and instability. For the second protein, 3EWH, the RMSF values for the complexed and uncomplexed Ca atoms are also presented in Figure 2. Most residues had RMSF values below 3 Å, suggesting stability, but certain amino acids showed high instability, with RMSF values ranging from 5 to 9.9 Å due to their large movements. A comparison of the residue stability between the two proteins reveals that the residues interacting with ligand **291** are the most stable in both proteins.

#### 2.3.3. Radius of Gyration and Solvent-Accessible Surface Area Parameter

**Figure 3:**
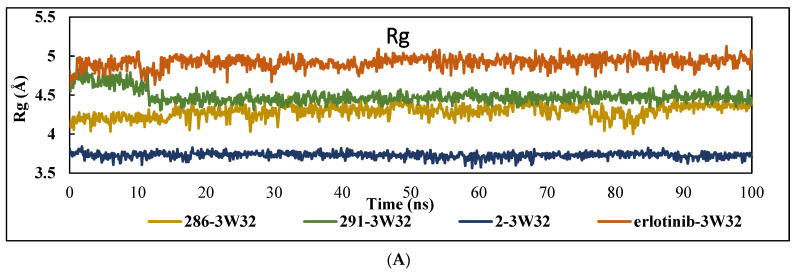

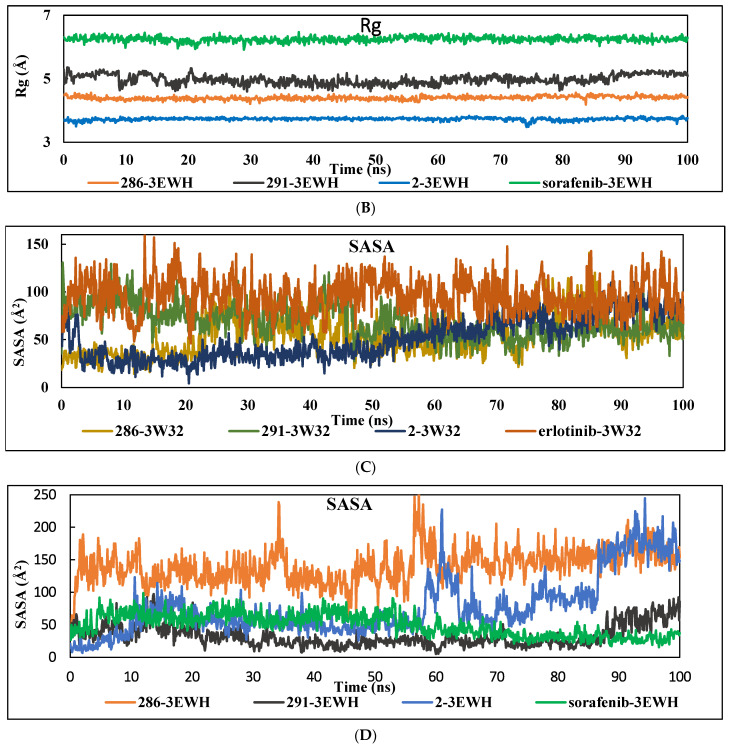
Rg and SASA graphs for the studied protein–ligand complexes with (**A**,**C**) for 2-EGFR, 286-EGFR, 291-EGFR, and Erlotinib-EGFR; (**B**,**D**) for 2-VEGFR2, 286-VEGFR2, 291-VEGFR2, and Sorafenib-VEGFR2.

The Rg provides a measure of the mass-weighted root mean square distance of a set of atoms from their common center of mass. This indicator is essential for assessing the stability of a protein–ligand complex during a molecular dynamics simulation. As shown in Figure 3, EGFR and VEGFR2 proteins bound to reference drugs show higher Rg values, with an average of 4.9 ± 0.07 Å for EGFR and 6.24 ± 0.07 Å for VEGFR2. These complexes therefore adopt a more extended conformation. On the other hand, ligand 291 bound to both proteins also showed relatively high Rg values, suggesting an expansive structure of the complexes formed. In contrast, ligand **2** showed lower Rg values in both proteins, with a similar mean of 3.73 ± 0.03 Å. These lower values suggest that the complexes formed with ligand **2** are more compact or globular as they show less variation in Rg. To evaluate the stability and structural compactness of the proteins, the solvent-accessible surface area (SASA) parameter was also taken. As shown in Figure 3, EGFR and its complexes were stable throughout the simulation, with mean values ranging from 50.79 to 95.4 Å^2^. In contrast, the four complexes of VEGFR2 formed with its ligand showed SASA values ranging from 49.64 to 140.97 Å^2^, where the 2-3EWH complex showed notable instability.

#### 2.3.4. Protein–Ligand Contacts

To gain deeper insight into the mechanism of action of the interactions within the complexes, we conducted a detailed analysis of the protein–ligand contacts. This study aimed to evaluate the types of interactions and identify the key residues that contribute to the stability of the complexes throughout the simulation.

**Figure 4:**
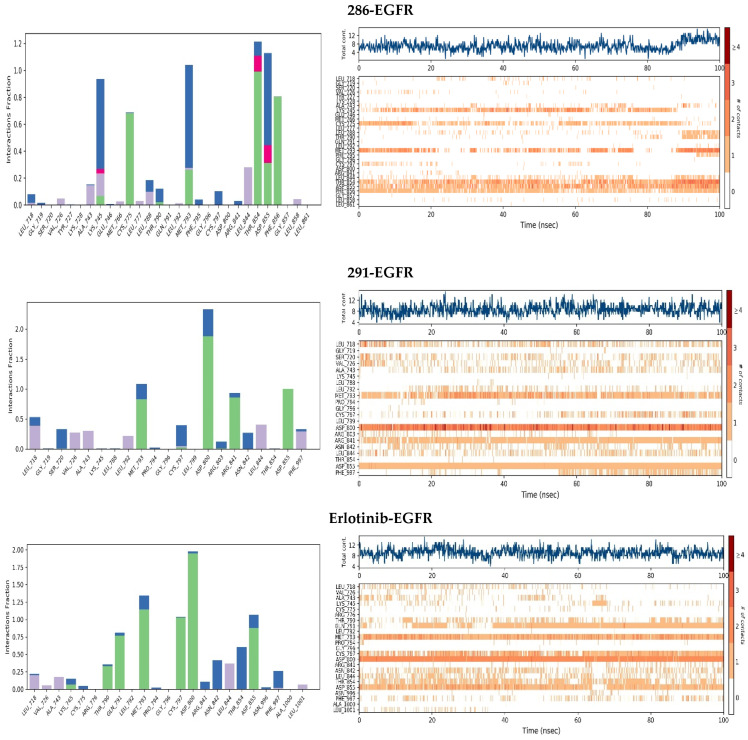

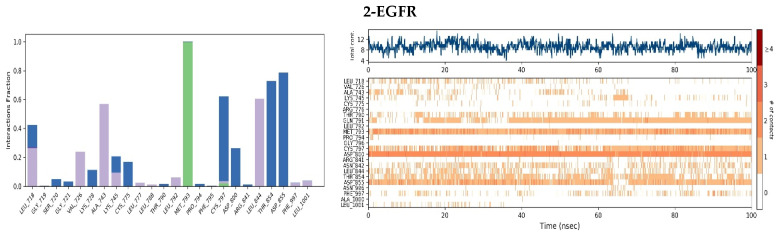
Protein–ligand interactions for the ligands studied with EGFR. Purple: hydrophobic bonds; blue: water–bridge; green: hydrogen bonds; pink: ionic bonds.

**Figure 5:**
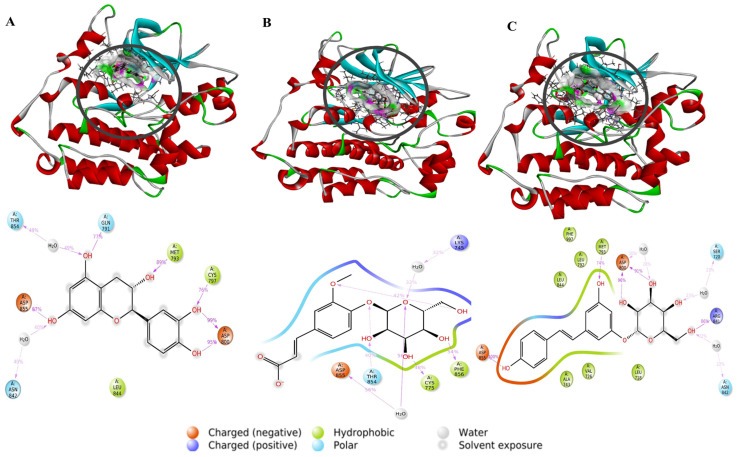
The 3D and 2D viewing of the protein–ligand interactions of 2-EGFR (**A**), 286-EGFR (**B**), and 291-EGFR (**C**).

As revealed in Figure 4 and Figure 5, the residues responsible for this docking score and the types of interactions carried out, as well as the continuity of the stability of the interactions in all the complexes, reveal that the most stable complex for the EGFR protein is complex 291-EGFR. This complex remains stable throughout the simulation trajectory due to hydrogen bonds with residues ASP800, ASP855, MET793, and ARG841, exhibiting a contact fraction between 0.9 and 1.8. When comparing these results with the reference drug, we observed that EGFR interacts with sorafenib through hydrogen bonds, hydrophobic interactions, and water bridges. In contrast, the stability of the erlotinib-EGFR complex during the 100 ns simulation was primarily due to hydrogen bonds with key residues ASP800, MET793, CYS797, and ASP855. The other complexes were also found to establish hydrogen, hydrophobic, and water–bridge interactions. In particular, the 286-EGFR complex also forms ionic interactions. However, their stability is essentially based on a predominant interaction with a key residue: ASP855 for the 286-EGFR complex and MET793 for the 2-EGFR complex. Several studies have highlighted the importance of these amino acids in the context of EGFR inhibition, emphasizing their role in hydrogen and hydrophobic interactions with ligands. These key residues are essential for stabilizing the binding between the inhibitor and the receptor, thereby increasing the affinity and efficacy of the drug.

**Figure 6:**
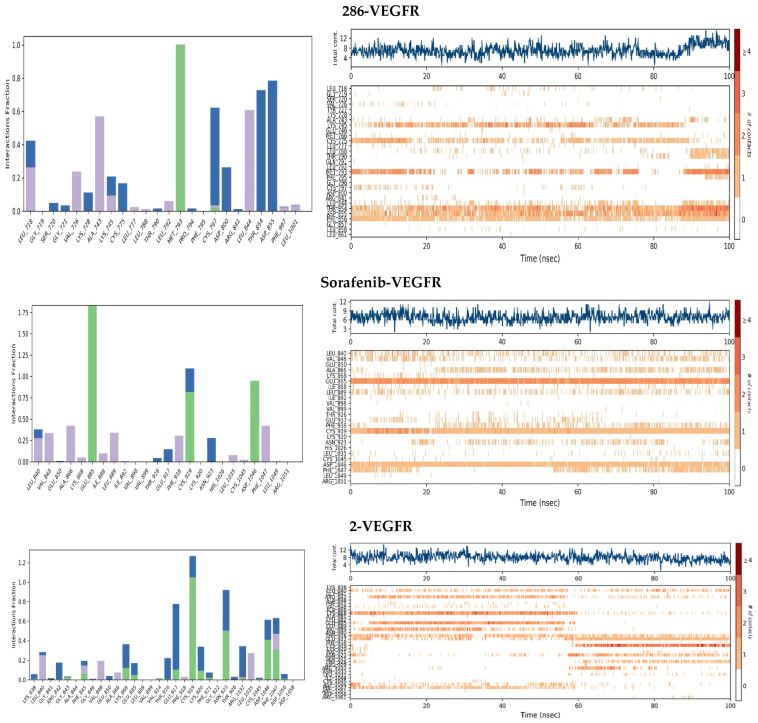

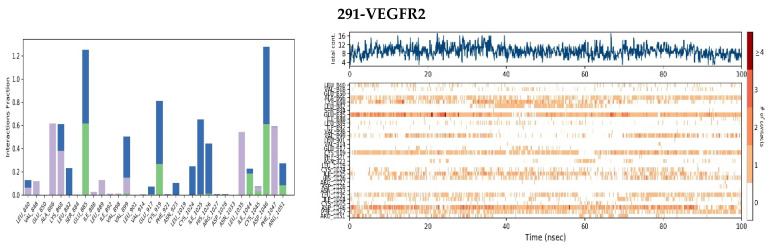
Protein–ligand interactions for the ligands studied with VEGFR2. Purple: hydrophobic bonds; blue: water–bridge; green: hydrogen bonds; pink: ionic bonds.

**Figure 7:**
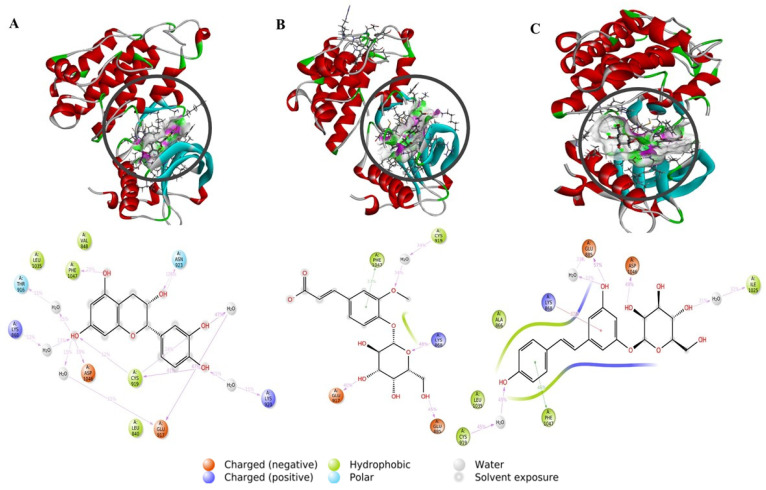
The 3D and 2D viewing of the protein–ligand interactions of 2-EGFR (**A**), 286-EGFR (**B**), and 291-EGFR (**C**).

For the VEGFR2 protein, all the complexes were found to be bound by hydrogen, hydrophobic, and water–bridge interactions, as illustrated in Figure 6 and Figure 7. However, the stability of the complex bound with erlotinib is mainly ensured by hydrogen interactions with the key residues GLU885, CYS919, and ASP1046, with a contact fraction of between 0.8 and 1.8. Compared with the other complexes, the 291-VEGFR2 complex proved to be the most stable throughout the simulation. This stability was due to hydrogen interactions and water bridges formed with the key residues GLU885, CYS919, and ASP1046, in addition to interactions with the **291** ligands. In contrast, the other complexes showed significant instability throughout the simulation. During this study, it was found that ferulic acid 4-O-glucoside, catechin, and resveratrol 3-glucoside possess remarkable antiangiogenics power. These compounds simultaneously inhibit VEGFR2 and EGFR receptor tyrosine kinases while displaying promising pharmacokinetic properties. According to our data, these polyphenols are prevalent in the *Ajuga iva* L. plant, highlighting its significant effectiveness against cancer. Furthermore, *Ajuga iva* L. has demonstrated its ability to inhibit both the VEGFR2 and EGFR receptors, which are critical in regulating multiple processes affecting both normal and cancer cell functions, including tumor growth, angiogenesis, cell proliferation, and survival. This dual inhibition not only underscores the plant’s potential as an anticancer agent but also suggests that it may contribute to a multifaceted approach in cancer treatment by targeting the key pathways involved in tumorigenesis. Many studies have demonstrated that *A. iva* L. exhibits a range of properties, such as antidiabetic, antioxidant, antimicrobial, anti-hypercholesterolemia, and insecticidal [30]. The health benefits of this plant are attributed to its high polyphenol content, which is recognized for its substantial impact on human health [31]. In our study, resveratrol 3-glucoside stands out for its great potential to be used as a candidate for the treatment of angiogenesis thanks to its effective inhibitory capacities and beneficial properties.

## 3. Material and Methods

### 3.1. Data Set

In this study, we collected 386 phytocompounds from 22 plants traditionally known to the Moroccan population for their anticancer properties [32]. However, despite their traditional use, these plants have not yet been thoroughly investigated through scientific research to formally evaluate their potential as cancer treatments. While they have been examined in previous studies for their antibacterial, antifungal, and antioxidant effects, there is still a notable lack of specific evaluations of their efficacy as anticancer agents. These 22 plants are derived from six different plant families, particularly Amaranthaceae, Asteraceae, Fabaceae, Lamiaceae, Pinaceae, and Apiaceae, which are found in different regions within Morocco [30,33,34,35,36,37,38,39,40,41,42,43,44,45,46,47,48,49,50,51,52,53,54,55,56,57,58,59,60]. The name and structure of the 22 plants, along with the parts used and their precise collection locations, are listed in Table S1 within the Supplementary Materials.

### 3.2. Molecular Docking

#### 3.2.1. Ligand Preparation

All 3D ligand structures were prepared using the LigPrep workflow available within Maestro 2020 (Schrodinger, New York, NY, USA). The ionization states of the ligands were generated at a pH of 7.0 ± 2.0, and the energy of the ligands was minimized using the optimized potential for liquid simulation force field (FF) OPLS3e [61]. The LigPrep interface was also used for desalting and tautomer generation, as well as to produce relevant stereoisomers set at a maximum of 32 conformers per ligand.

#### 3.2.2. Protein Preparation

As targets, two proteins were selected for these computational studies: EGFR (PDB: 3W32) [62] and VEGFR-2 (PDB: 3EWH) [63]. High-resolution protein crystal structures were extracted from the Protein Data Bank (PDB) (https://www.rcsb.org/, accessed on 20 January 2024) (r = 1.8 Å and 1.6 Å, respectively). Structures were then prepared using the ProtPrep workflow, which includes adding hydrogens, assigning bond orders, adding missing side chains (and loops), as well as H-bond assignments, removal of bulk water, and protein structure minimization using the OPLS3e FF [64,65].

#### 3.2.3. GridBox Generation 

The grid box defines the three-dimensional space within the protein binding pocket that the proposed ligands will survey. This enables numerous ligand conformations to efficiently sample a binding pocket and ultimately derive quantitative docking scores for analysis. The grid centers were determined by the initial positions of the co-crystallized ligands, which guided the selection of coordinates for the EGFR and VEGFR2 receptors; specifically, the grid center for the EGFR receptor was set at (x = −51.396, y = −0.627, z = −18.744), while, for the VEGFR2 receptor, it was set at (x = 15.555, y = −5.493, z = 9.661). The dimensions of the grid box are the same for both receptors, with values of 30 × 30 × 30 Å in the x, y, and z directions.

#### 3.2.4. Receptor–Ligand Docking 

Molecular docking was used to identify the most effective natural compounds that target EGFR and VEGFR2, while structure-based virtual screening was conducted with Schrödinger software (Version 12.5.139). The approach started with high-throughput virtual screening (HTVS) to conduct an initial assessment of the complete ligand library. This was followed by more detailed and refined docking analyses using Glide’s standard precision (SP) and extra precision (XP) docking workflows [66]. The marketed 4-aminoquinazoline erlotinib (Tarceva^TM^) and urea-based kinase inhibitor sorafenib (Nexavar^TM^) were utilized as positive controls for EGFR and VEGFR2, respectively. These successive enrichment-based filtering docking workflows ensure that the most promising ligands, in terms of binding affinity and potential biological activity, proceed to the next step and are accurately identified and prioritized going from HTVS to SP and finally to XP.

### 3.3. ADMET Prediction

The top ligands selected on the basis of their docking scores were then evaluated for their predicted ADMET (Adsorption, Distribution, Metabolism, Excretion, and Toxicity) properties using SwissADME (http://www.swissadme.ch/, accessed on 25 January 2024) and pkCSM (https://biosig.lab.uq.edu.au/pkcsm/, accessed on 25 January 2024) web resources. This secondary screening step was performed to ensure that the ligands selected not only had high predicted affinities (i.e., docking scores) but also favorable pharmacokinetic and safety profiles. The drug-likeness of a compound was predicted through the use of Lipinski’s rule of 5 (Ro5), developed to define a set of physicochemical properties to predict oral bioavailability. The Ro5 states that oral small molecules likely possess H-bond donors lower than 5, H-bond acceptors lower than 10, a molecular weight under 500 Da, and a calculated Log P not exceeding 5 [67].

### 3.4. Molecular Dynamics

In our study, we aimed to evaluate the stability of the ligands that are secure and multi-targeted against both receptors using Schrödinger Maestro software (Version 12.5.139) over a duration of 100 ns [68]. The receptors were prepared by eliminating overlapping water molecules and solvating the molecular system with TIP3P water molecules. To neutralize the system, Na^+^ and Cl^−^ ions were added, resulting in a salt concentration of 0.15 M. The OPLS3e force field was then employed to assign parameters for the protein [69,70]. Temperature and pressure conditions were stabilized using an isothermal–isobaric ensemble with the Nose–Hoover thermostat and Martyna–Tobias–Klein barostat, which are the default protocols [71]. Following these preparations, the systems were subjected to NVT equilibration for 1 ns, followed by NPT equilibration at 310.5 K and 1.01 bar for a total simulation duration of 100 ns. Throughout the simulation, trajectories were recorded at intervals of 100 ps, generating approximately 1000 frames [72]. The results were analyzed using root mean square deviation (RMSD), root mean square fluctuation (RMSF), radius of gyration (Rg), solvent-accessible surface area (SASA), and protein–ligand contact to assess variations and flexibility in the molecular structures over time.

## 4. Conclusions

Morocco’s native plants have historically shown strong and broad medicinal utility across various diseases, including cancers. Commonly used by the Moroccan population to treat a variety of ailments, these herbs are distinguished by their high content of polyphenols, known for their beneficial properties on human health. In this study, we explored the anticancer activity of 386 natural compounds extracted from 22 plants from different regions of Morocco. This exploration was carried out by targeting angiogenesis-associated receptor tyrosine kinases, notably EGFR and VEGFR2, using virtual screening. The results highlighted that three specific ligands, catechin, ferulic acid 4-O-glucoside, and resveratrol 3-glucoside, possessed remarkable properties and significant potential for inhibiting angiogenesis, a crucial process in cancer development. Their docking scores were −10.959, −10.002, and −10.187 kcal/mol, respectively, for VEGFR2, and −9.463, −10.288, and −12.675 kcal/mol, respectively, for EGFR. Notably, these three polyphenols are present in the plant *Ajuga iva* L., demonstrating its promising potential as an anticancer agent. To strengthen our conclusions, it is important to recognize the study’s limitations, especially the lack of experimental validation for the computational methods used. By incorporating in vitro and in vivo studies and taking a multidisciplinary approach, we can enhance the credibility of our results and support their use in clinical settings. Our findings set the stage for future research that could confirm the anticancer properties of *Ajuga iva* L. and help to develop new therapeutic strategies using underutilized plant resources. A thorough evaluation will help to determine their true effectiveness and potential as treatments.

## Figures and Tables

**Table 1:** Docking score of compounds docked to EGFR and VEGFR2 receptors.

VEGFR2 (3EWH)			EGFR (3W32)		
Compound		Docking Score (kcal/mol)	Compound		Docking Score (kcal/mol)
Apigenin 7-*O*-(6″-malonyl-apiosyl-glucoside)	293	−15.2	Apigenin 7-*O*-(6″-malonyl-apiosyl-glucoside)	293	−15.3
Hesperidin	17	−14.8	Hesperidin	17	−14.9
Luteolin-hexoside	320	−13.3	Resveratrol 3-Glucoside	291	−12.6
Epigallocatechin gallate	288	−12.9	Apigenin−7-(2-*O*-apiosylglucoside)	292	−12.6
Dihydroxybenzoic acid	110	−12.8	Catechin−7-*O*-glucoside	283	−12.6
Caffeoylglucaric acid	100	−12.8	Rutin	3	−12.3
Catechin−7-*O*-glucoside	283	−12.7	Naringin	16	−11.9
Luteolin-dihexoside	319	−11.0	Caffeoylglucaric acid	100	−11.7
Luteolin-glucuronide	321	−11.0	Isochlorogenic acid b	109	−11.1
Epicatechin	10	−10.9	Isochlorogenic acid C	111	−11.1
Catechin	2	−10.9	Myricetin	276	−10.8
5-*O*-Caffeoylquinic acid	331	−10.9	Rhamnetin	282	−10.8
Chlorogenic acid	4	−10.8	Citronellyl Acetate	328	−10.7
Apigenin−7-(2-*O*-apiosylglucoside)	292	−10.8	Quercetin	7	−10.3
Salvianolic acid B	324	−10.8	Ferulic acid 4-*O*-glucoside	286	−10.2
Apigenin-glucuronide	323	−10.7	Salvianolic acid B	324	−10.2
Salvianolic acid A	326	−10.6	4-*O*-Caffeoylquinic acid	106	−10.1
Apigenin-dihexoside	322	−10.5	Luteolin-dihexoside	319	−10.1
Quercetin	7	−10.4	Salvianolic acid A	326	−10.1
Myricetin	276	−10.2	Luteolin-hexoside	320	−9.6
Harpagid	284	−10.2	Kaempferide	296	−9.5
Resveratrol 3-Glucoside	291	−10.1	Feruloylquinic acid	105	−9.5
Rosmarinic acid	325	−10.1	Epigallocatechin gallate	288	−9.5
Naringin	16	−10.0	Chlorogenic acid	4	−9.4
Ferulic acid 4-*O*-glucoside	286	−10.0	Catechin	2	−9.4
Vanillic acid glucoside	298	−9.8	5-*O*-caffeoylquinic acid	331	−9.4
Luteolin	22	−9.7	Luteolin-glucuronide	321	−9.3
Sorafenib		−9.7	Erlotinib		−9.2

**Table 2:** Physicochemical properties of ligands docked in both receptors.

Compound	MW	LogP	HbA	HbD	Lipinski
**2**	290.27	1.54	6	5	Yes
**3**	610.52	−1.68	16	10	No
**4**	354.31	−0.64	8	6	Yes
**7**	302.23	1.98	7	5	Yes
**10**	290.27	1.54	6	5	Yes
**16**	580.53	−1.16	14	8	No
**17**	610.56	−1.15	15	8	No
**100**	372.28	−2.33	9	8	Yes
**105**	368.33	−0.34	8	5	Yes
**106**	354.31	−0.64	8	6	Yes
**109**	516.45	1.03	11	7	No
**110**	448.38	0.48	11	7	No
**111**	516.45	1.03	11	7	No
**276**	318.23	1.69	8	6	Yes
**282**	316.26	2.29	7	4	Yes
**283**	452.41	−0.98	11	8	No
**284**	364.34	−3.46	10	7	No
**286**	356.32	−1.02	8	5	Yes
**288**	458.37	2.23	11	8	No
**291**	390.38	0.44	8	6	Yes
**292**	564.49	−1.48	14	8	No
**293**	650.54	−1.46	16	8	No
**296**	300.26	2.58	6	3	Yes
**298**	330.28	−1.42	8	5	Yes
**319**	610.52	−2.77	16	10	No
**320**	448.38	−0.24	11	7	No
**321**	462.36	−0.15	11	7	No
**322**	594.52	−2.47	15	9	No
**323**	446.36	0.14	10	6	No
**324**	718.62	3.33	14	9	No
**325**	360.31	1.76	7	5	Yes
**326**	494.45	3.34	9	7	Yes
**328**	538.46	2.72	10	6	No
**331**	354.31	−0.64	8	6	Yes
**22**	286.23	2.28	1	6	Yes

MW: molecular weight (g/mol); HbA: number of H-bond acceptors; HbD: number of H-bond donors.

**Table 3:** The pharmacokinetic properties of anchored ligands.

	WS	Caco2	IAb	BBB	CYP2D6	CYP3A4	CYP1A2	CYP2C19	CYP2C9	T.C.	ROCT2
**2**	−2.99	−0.29	67.96	−1.16	No	No	No	No	No	0.24	No
**4**	−2.62	−0.98	1.79	−1.70	No	No	No	No	No	0.30	No
**7**	−3.26	0.73	69.79	−1.45	No	No	Yes	No	No	0.60	No
**10**	−2.98	−0.44	72.13	−1.18	No	No	No	No	No	0.23	No
**22**	−3.08	1.00	78.80	−2.73	No	No	Yes	No	No	0.66	No
**100**	−2.88	−1.21	0	−2.45	No	No	No	No	No	0.15	No
**105**	−2.03	−0.49	33.32	−1.33	No	No	No	No	No	0.41	No
**106**	−1.99	−0.78	8.82	−1.91	No	No	No	No	No	0.32	No
**276**	−3.03	0.23	63.00	−1.72	No	No	Yes	Yes	Yes	0.52	No
**282**	−3.44	0.56	80.49	−1.44	No	No	Yes	No	No	0.61	No
**286**	−2.46	−0.65	26.01	−1.65	No	No	No	No	No	0.26	No
**291**	−3.78	0.18	45.49	−1.34	No	No	No	No	No	0.28	No
**296**	−3.41	1.03	80.34	−1.31	No	No	Yes	Yes	Yes	0.71	No
**298**	−2.46	−0.56	29.18	−1.68	No	No	No	No	No	0.68	No
**325**	−3.42	0.01	47.96	−1.54	No	No	No	No	No	0.36	No
**326**	−2.91	−0.26	48.06	−1.97	No	No	Yes	No	No	0.12	No
**331**	−2.22	−0.57	9.03	−1.89	No	No	No	No	No	0.36	No

WS: water solubility; IAb: intestinal absorption; BBB: blood–brain barrier permeability; T.C.: total clearance (log mL/min/kg); ROCT2: renal organic cation transporter2 substrate.

**Table 4:** Prediction of the toxicity of the natural products studied that adhere to Lipinski’s rules.

	AMES	Max. D	hERG I	hERG II	LD50	LOAEL	Hepatotoxicity	S.S.
**2**	No	0.76	No	No	1.82	2.97	No	No
**4**	No	1.05	No	No	1.86	3.90	No	No
**7**	No	0.99	No	No	2.08	2.77	No	No
**10**	No	0.60	No	No	2.03	2.01	No	No
**22**	No	0.83	No	No	2.23	2.05	No	No
**100**	No	0.56	No	No	2.47	5.12	No	No
**105**	No	0.64	No	No	2.50	3.67	No	No
**106**	No	0.13	No	No	2.67	3.88	No	No
**276**	No	0.76	No	No	2.57	3.04	No	No
**282**	Yes	0.92	No	No	2.42	2.83	No	No
**286**	No	0.52	No	No	2.94	3.69	No	No
**291**	No	0.45	No	No	3.70	3.66	No	No
**296**	No	0.90	No	No	2.24	2.41	No	No
**298**	No	0.85	No	No	2.76	3.78	Yes	No
**325**	No	0.50	No	No	2.47	3.54	No	No
**326**	No	0.51	No	Yes	2.61	4.65	No	No
**331**	No	0.26	No	No	2.93	3.64	No	No

Max. D: maximum tolerated dose; LD50: oral rat acute toxicity; LOAEL: oral rat chronic toxicity; S.S.: skin sensitization.

**Table 5:** The leading ligands and their chemical structures.

Compound Name	Compound ID	Structure
Catechin	**2**	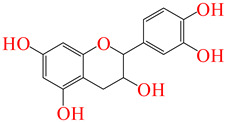
Ferulic acid 4-*O*-glucoside	**286**	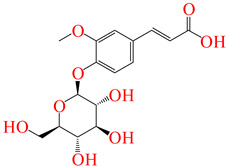
Resveratrol 3-Glucoside	**291**	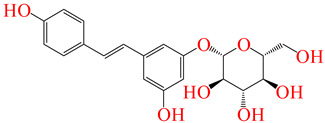

## Data Availability

Data are contained within the article.

## Supplementary Materials

The following supporting information can be downloaded at https://www.mdpi.com/article/10.3390/ph17111528/s1, Table S1: Detailed Information on Plant Species, Botanical Families, Chemical Composition, Collection Locations, and Harvested Plant Parts; Table S2: 2D Structures of Chemical Compounds Extracted from Plants.
