# Supplementary material for: Exploring Moroccan Medicinal Plants for Anticancer Therapy Development Through In Silico Studies
Source: Pharmaceuticals (Basel). 2024 Nov 13;17(11):1528. doi: 10.3390/ph17111528 (PMC11597486; doi:10.3390/ph17111528)
Supplement: Supplementary file 1 [file pharmaceuticals-17-01528-s001.zip › pharmaceuticals-3277355-supplementary.pdf]

Exploring Moroccan Medicinal Plants for Anticancer Therapy Development Through In Silico Studies

Amal Bouribab [1], El Mehdi Karim [1], Meriem Khedraoui [1], Oussama Abchir [1], Abdelkbir Errougui [1], Yasir S. Raouf [2], Abdelouahid Samadi [2,*] and Samir Chtita [1,*]

[1] Laboratory of Analytical and Molecular Chemistry, Faculty of Sciences Ben M'Sik, Hassan II University of Casablanca, Casablanca 20600, Morocco; bouribabamal@gmail.com (A.B.); 2013karim.mehdi@gmail.com (E.M.K.); meriemkhedraoui5@gmail.com (M.K.); oussamaabchir12@gmail.com (O.A.); a_errougui@yahoo.fr (A.E.)

[2] Department of Chemistry, College of Science, United Arab Emirates University, Al Ain P.O. Box 15551, United Arab Emirates; yasir.raouf@uaeu.ac.ae

* Correspondence: authors: samadi@uaeu.ac.ae (A.S.); samirchtita@gmail.com (S.C.)

Supplementary material

Table S1: Detailed Information on Plant Species, Botanical Families, Chemical Composition, Collection Locations, and Harvested Plant Parts

Botanical family	Plant	Compound	N°	Smiles	Extract	Part of the Plant and Collection Zone
Amaranthaceae	*Anabasis aretioides* L.	Gallic acid	1	OC(C1=CC(O)=C(O)C(O)=C1)=O	Cold maceration extract	Aerial parts, Roots and seeds from the region of Errachidia [1]
		Catechin	2	OC1=CC2=C(C[C@@H](O)[C@H](C3=CC=C(O)C(O)=C3)O2)C(O)=C1		
		Rutin	3	OC1=CC(O)=C(C2=C1)C(C(O[C@@H]3[C@@H](O)[C@H](O)[C@@H](O)[C@H](CO[C@H]4O[C@@H](C)[C@H](O)[C@@H](O)[C@@H]4O)O3)=C(O2)C5=CC=C(O)C(O)=C5)=O		
		Chlorogenic acid	4	OC1=CC=C(C=C1O)/C=C/C(O[C@H]2[C@H](O)[C@@H](O)C[C@@](O)(C(O)=O)C2)=O		
		Vanillic acid	5	OC1=C(OC)C=C(C(O)=O)C=C1		
		Caffeic acid	6	OC1=CC(/C=C/C(O)=O)=CC=C1O		
		Quercetin	7	OC1=CC(O)=C(C(C(O)=C(C2=CC=C(O)C(O)=C2)O3)=O)C3=C1		
		Syringic acid	8	COC1=CC(C(O)=O)=CC(OC)=C1O		
		P-hydroxybenzoïc acid	9	OC1=CC=C(C(O)=O)C=C1		
		Epicatechin	10	O[C@H]1[C@H](C2=CC=C(O)C(O)=C2)OC3=C(C1)C(O)=CC(O)=C3		
		Coumaric acid	11	OC1=CC=C(/C=C/C(O)=O)C=C1		
		Salicylic acid	12	OC1=C(C(O)=O)C=CC=C1		
		Catechol	13	OC1=C(O)C=CC=C1		
		Pyrogallol	14	OC1=C(O)C(O)=CC=C1		
		Ferulic acid	15	COC1=C(O)C=CC(/C=C/C(O)=O)=C1		
		Naringin	16	OC1=CC=C(C=C1)[C@H]2CC(C3=C(O2)C=C(C=C3O)O[C@@H]4O[C@H](CO)[C@@H](O)[C@H](O)[C@H]4O[C@@H]5[C@@H](O)[C@H](O)[C@@H](O)[C@H](C)O5)=O		

		Hesperidin	17	OC1=C(OC)C=CC([C@H]2CC(C3=C(O2)C=C(C=C3O)O[C@H]4[C@H](O)[C@@H](O)[C@H](O)[C@H](O4)CO[C@@H]5[C@@H](O)[C@H](O)[C@@H](O)[C@H](C)O5)=O)=C1		
		3-hydroxycinnamic acid	18	OC(/C=C/C1=CC(O)=CC=C1)=O		
		4-hydroxycinnamic acid	11	OC1=CC=C(/C=C/C(O)=O)C=C1		
		3,4-dihydroxybenzoic acid	20	OC1=C(O)C=CC(C(O)=O)=C1		
		3-hydroxybenzoic acid	21	OC1=CC=CC(C(O)=O)=C1		
		Luteolin	22	O=C1C2=C(O)C=C(O)C=C2OC(C3=CC(O)=C(O)C=C3)=C1		
		Naringenin	23	OC1=CC=C(C=C1)[C@H]2CC(C3=C(O2)C=C(O)C=C3O)=O		
		Hesperetin	24	OC1=CC2=C(C(C[C@H](C3=CC=C(OC)C(O)=C3)O2)=O)C(O)=C1		
	Haloxylon scoparium L.	Decanoic acid, methyl ester	25	CCCCCCCCCC(OC)=O	Dichloromethane extract	Aerial part and root part in the region of Figuig in Moroccan Sahara [2]
		Heptandioic acid, dimethyl ester	26	COC(CCCCCC(OC)=O)=O		
		4-Ketopelarrgonic acid methyl ester	27	CCCCCC(CCC(OC)=O)=O		
		Methyl 8-oxononanoate	28	CC(CCCCCCC(OC)=O)=O		
		Undecanoic acid, methyl ester	29	CCCCCCCCCCC(OC)=O		
		Nonanoic acid, 9-oxo-, methyl ester	30	COC(CCCCCCCC=O)=O		
		Octanedioic acid, dimethyl ester	31	COC(CCCCCCC(OC)=O)=O		
		Dodecanoic acid, methyl ester	32	CCCCCCCCCCCC(OC)=O		
		Nonanedioic acid, dimethyl ester	33	COC(CCCCCCCC(OC)=O)=O		
		Tridecanoic acid, methyl ester	34	CCCCCCCCCCCCC(OC)=O		
		Decanedioic acid, dimethyl ester	35	COC(CCCCCCCCC(OC)=O)=O		
		Tetradecanoic acid, methyl ester	36	CCCCCCCCCCCCCC(OC)=O		
		Undecanedioic acid, dimethyl ester	37	COC(CCCCCCCCCC(OC)=O)=O		
		Pentadecanoic acid, methyl ester	38	CCCCCCCCCCCCCCC(OC)=O		
		Dodecanedioic acid, dimethyl ester	39	COC(CCCCCCCCCCC(OC)=O)=O		
		Dodecanedioicacid, dimethyl ester	39	COC(CCCCCCCCCCC(OC)=O)=O		
		15-methylhexadecanoic acid, methyl ester	40	CC(CCCCCCCCCCCCC(OC)=O)C		
		Hexadecanoic acid, methyl ester	41	CCCCCCCCCCCCCCCC(OC)=O		
		Heptadecanoic acid, methyl ester	42	CCCCCCCCCCCCCCCCC(OC)=O		
		Γ-dodecalactone	43	CCCCCCCC[C@@H](O1)CCC1=O		
		10,13-octadecadienoic acid, methyl ester	44	CCCC/C=C/C/C=C/CCCCCCCCC(OC)=O		

		9,12-octadecadienoic acid, methyl ester	45	CCCCC/C=C\C/C=C\CCCCCCCC(OC)=O		
		12,15-octadecadienoic acid, methyl ester	46	CC/C=C/C/C=C/CCCCCCCCCCC(OC)=O		
		9,12,15-octadecatrienoic acid, methyl ester	47	CC/C=C/C/C=C/C/C=C/CCCCCCCC(OC)=O		
		13-octadecenoic acid, methyl ester	48	CCCC/C=C/CCCCCCCCCCCC(OC)=O		
		9-octadecenoic acid, methyl ester	49	CCCCCCCC/C=C/CCCCCCCC(OC)=O		
		11-octadecenoic acid, methyl ester	50	CCCCCC/C=C/CCCCCCCCCC(OC)=O		
		Octadecanoic acid, methyl ester	51	CCCCCCCCCCCCCCCCCC(OC)=O		
		Nonadecanoic acid, methyl ester	52	CCCCCCCCCCCCCCCCCCC(OC)=O		
		6,9,15-octadecatrienoic acid, methyl ester	53	CC/C=C\CCCC/C=C\C/C=C\CCCCC(OC)=O		
		6,9,12-octadecatrienoic acid, methyl ester	54	CCCCC/C=C/C/C=C/C/C=C/CCCCC(OC)=O		
		7,10,13-octadecatrienoic acid, methyl ester	55	CCCCC=CCC=CCC=CCCCCCC(OC)=O		
		Eicosanoic acid, methyl ester	56	CCCCCCCCCCCCCCCCCCCC(OC)=O		
		Heneicosanoic acid, methyl ester	57	CCCCCCCCCCCCCCCCCCCCC(OC)=O		
		Docosanoic acid, methyl ester	58	CCCCCCCCCCCCCCCCCCCCCC(OC)=O		
		Tricosanoic acid, methyl ester	59	CCCCCCCCCCCCCCCCCCCCCCC(OC)=O		
		Tetracosanoic acid, methyl ester	60	CCCCCCCCCCCCCCCCCCCCCCCC(OC)=O		
		Pentacosanoic acid, methyl ester	61	CCCCCCCCCCCCCCCCCCCCCCCCC(OC)=O		
		Docosanedioic acid, dimethyl ester	62	COC(CCCCCCCCCCCCCCCCCCCCC(OC)=O)=O		
		Hexacosanoic acid, methyl ester	63	CCCCCCCCCCCCCCCCCCCCCCCCCC(OC)=O		
		Acetamide, N-methyl-N-(2-phenylethyl)-	64	CC(N(CCC1=CC=CC=C1)C)=O	Ethyl acetate extract, butanolic extract and aqueous extract.	Aerial part and Root part in the region of Figuig in Moroccan sahara [2]
		4-(2-(N-methylacetamido)ethyl)phenyl acetate	65	CC(OC1=CC=C(CCN(C)C(C)=O)C=C1)=O		
		Acetamide, N-[2-(acetoxy)-2-phenylethyl]-	66	CC(NC[C@H](OC(C)=O)C1=CC=CC=C1)=O		
		6,7-dimethoxy-4-methylquinoline	67	CC1=CC=NC2=CC(OC)=C(OC)C=C12		
		N-(4-Hydroxyphenethyl)acetamide	68	CC(NCCC1=CC=C(O)C=C1)=O		
		9H-Pyrido[3,4-b]indole, N-acetyl, 1-methyl-	69	O=C(C)N1C2=C(C)N=CC=C2C3=CC=CC=C13		
		Acetamide, N-[2-[4-(acetyloxy)−3-methoxyphenyl]ethyl]-	70	CC(NCCC1=CC=C(OC(C)=O)C(OC)=C1)=O		
		3-(2-N-Acetyl-N-methylaminoethyl)indol	71	CC(N(CCC1=CNC2=C1C=CC=C2)C)=O		
		4-[1-Acetoxy-2-(diacetylamino)ethyl]phenyl acetate	72	CC(N(C(C)=O)C[C@H](OC(C)=O)C1=CC=C(OC(C)=O)C=C1)=O		
		Tryptamine, 2 acetyl	73	NCCC1=C(C(C)=O)NC2=C1C=CC=C2		

		1-Acetylamino-2-(4-acetoxy-3-hydroxyphenyl)ethane	74	OC1=CC(CCNC(C)=O)=CC=C1OC(C)=O		
		1-acetylamino-2-(4-acetoxy-3-hydroxyphenyl)ethane	74	OC1=CC(CCNC(C)=O)=CC=C1OC(C)=O		
		1,2,3,4-Tetrahydroisoquinolin, 1-methyl-2-acetyl-6-acetoxy-7-methoxy-	75	C[C@H]1C2=C(CCN1C(C)=O)C=C(OC(C)=O)C(OC)=C2		
		Isoquinolin-1-carboxylicacid, 1,2,3,4-tetrahydro-2-acetyl	76	O=C(O)[C@@H]1N(C(C)=O)CCC2=C1C=CC=C2		
		N-methyltryptamine, 2 acetyl	77	CNCCC1=C(C(C)=O)NC2=C1C=CC=C2		
		2-acetyl-1-methyl-1,2,3,4-tetrahydroisoquinoline-6,7-diyl diacetate	78	C[C@@H]1N(C(C)=O)CCC2=C1C=C(OC(C)=O)C(OC(C)=O)=C2		
		D-glucose, 2,3,4,5,6-pentaacetate	79	O=C[C@H](OC(C)=O)[C@@H](OC(C)=O)[C@H](OC(C)=O)[C@H](OC(C)=O)COC(C)=O		
		A-D-Glucopyranose pentaacetate	80	CC(O[C@@H]1[C@H](OC(C)=O)[C@@H](OC(C)=O)[C@H](OC(C)=O)[C@@H](COC(C)=O)O1)=O		
		Alpha-D-Glucopyranoside, 1,3,4,6-tetra-O-acetyl-beta-D-fructofuranosyl, 2,3,4,6-tetraacetate	81	O=C(C)OC[C@@H](O[C@](COC(C)=O)1O[C@@H]2[C@H](OC(C)=O)[C@@H](OC(C)=O)[C@H](OC(C)=O)[C@@H](COC(C)=O)O2)[C@@H](OC(C)=O)[C@@H]1OC(C)=O		
		1,1,2-triacetoxyethane	82	CC(OCC(OC(C)=O)OC(C)=O)=O		
		A-D-Galactose pentaacetate	83	CC(OC[C@@H]1[C@H](OC(C)=O)[C@H](OC(C)=O)[C@@H](OC(C)=O)[C@@H](OC(C)=O)O1)=O		
		Lyxopyranose tetraacetate	84	CC(O[C@@H]1CO[C@@H](OC(C)=O)[C@H](OC(C)=O)[C@H]1OC(C)=O)=O		
Astraceae	*Anacyclus pyrethrum* L.	N-isobutyl-dodeca-2,4,8,10-tetraenamide	95	C/C=C\C=C/CC/C=C/C=C/C(NCC(C)C)=O	Maceration (ethanol exctract)	Roots, seeds, leaves, and capitula from the timahdite region of morocco [3]
		Sarcosine, N-(trifluoroacetyl)-, butyl ester	86	CCCCOC(CN(C(C(F)(F)F)=O)C)=O		
		N-isobutyl-2,4-octadiene-6- monoynamide	87	CC#C/C=C/C=C/C(NCC(C)C)=O		
		Levulinic acid	88	CC(CCC(O)=O)=O		
		Propanedioic acid	89	O=C(O)CC(O)=O		
		N-isobutyl-2,4-heptadiene-6- monoynamide	90	CC(CNC(/C=C/C=C/C#C)=O)C		
		Palmitic acid	91	CCCCCCCCCCCCCCCC(O)=O		
		Morphinan-6-One, 4,5.α.-Epoxy-3-Hydroxy-17-Methyl	92	CN1CC[C@]23[C@@H]4[C@H]1CC5=C2C(O[C@H]3C(CC4)=O)=C(O)C=C5		

		Cinnamic acid	93	O=C(O)/C=C/C1=CC=CC=C1		
		(2E,4E)-N-isobutylundeca-2,4-dien-8,10-diynamide	94	CC(CNC(/C=C/C=C/CCC#CC#C)=O)C		
		2,4-undecadiene-8,10-diyne-N-tyramide	151	C/C=C/C#CC#C/C=C/C=C/C=C/C(NCC(C)C)=O		
		N-isobutyl-dodeca-2,4,8,10-tetraenamide (Anacycline)	95	C/C=C\C=C/CC/C=C/C=C/C(NCC(C)C)=O		
		N-isobutyl-2,6,8-decatrienamide	96	C/C=C/C=C/CC/C=C/C(NCC(C)C)=O		
		(2E,4E)-N-(2-methylpropyl)deca-2,4-dienamide (Pellitorine)	103	CCCCC/C=C/C=C/C(NCC(C)C)=O		
		Tetradeca-2E-diny-8,10-diynoic acid IBA	150	OC1=CC=C(C=C1)CCNC(/C=C/C=C/CC/C=C/CCCCC)=O		
		Tetradeca-2E,4E, ne-trienoic-8,10-diynoic acid IBA	155	CCCC#CC#CCC/C=C/C=C/C(NCC(C)C)=O		
		Isovaleric acid	98	CC(CC(O)=O)C		
		Dodeca-2E,4E, ne-trienoic acid 4-hydroxyphenylethylamide	152	CCC/C=C/CC/C=C/C=C/C(NCCC1=CC=C(O)C=C1)=O		
		2,8-N-isobutyl-2,8-dodecadienamide	210	CCC/C=C/CCCC/C=C/C(=O)NCC(C)C		
		Tetradeca-2E,4E,8Etrienoic acid 4-hydroxyphenylethylamide	153	OC1=CC=C(C=C1)CCNC(/C=C/C=C/CC/C=C/CCCCC)=O		
		Citric acid	99	O=C(O)CC(C(O)=O)(O)CC(O)=O	Aqueous macerate extract	Roots Bin El Ouidan region, Morocco [4]
		Gallic acid	1	OC(C1=CC(O)=C(O)C(O)=C1)=O		
		Dihydroxybenzoic acid	110	C[C@H]1[C@H](O)[C@@H](O)[C@@H](O)[C@H](OC2=C(C3=CC=C(O)C(O)=C3)OC4=CC(O)=CC(O)=C4C2=O)O1		
		Dihydroxybenzoic acid glucoside	352	C1=CC(=C(C=C1O[C@H]2[C@@H]([C@H]([C@@H]([C@H](O2)CO)O)O)O)C(=O)O)O		
		Caffeoylglucaric acid	100	O=C([C@@](C(O)=O)(O)[C@@H](O)[C@H](O)[C@H](O)C(O)=O)/C=C/C1=CC=C(O)C(O)=C1		
		Gallic acid	1	OC(C1=CC(O)=C(O)C(O)=C1)=O		
		3,4-dihydroxybenzoic acid	20	OC1=C(O)C=CC(C(O)=O)=C1		
		Hydroxybenzoic acid glycerol	19	C1=CC=C(C(=C1)C(=O)OCC(CO)O)O		
		Chlorogenic acid	4	OC1=CC=C(C=C1O)/C=C/C(O[C@H]2[C@H](O)[C@@H](O)C[C@@](O)(C(O)=O)C2)=O		
		Dihydroxybenzoic acid glucuronide	355	C1=CC(=C(C=C1C(=O)O)OC2C(C(C(C(O2)C(=O)O)O)O)O)O		

		Pellitorine	103	CCCCC/C=C/C=C/C(NCC(C)C)=O		
		Dihydrocaffeic acid	104	O=C(O)CCC1=CC=C(O)C(O)=C1		
		Feruloylquinic acid	105	COC1=CC(/C=C/C(O[C@@H]2C[C@@](C(O)=O)(O)C[C@@H](O)[C@H]2O)=O)=CC=C1O		
		Cryptochlorogenic acid	106	O=C(O)[C@]1(O)C[C@@H](O)[C@@H](OC(/C=C/C2=CC=C(O)C(O)=C2)=O)[C@H](O)C1		
		Caffeic acid	6	OC1=CC(/C=C/C(O)=O)=CC=C1O		
		P-Coumaric acid	11	OC1=CC=C(/C=C/C(O)=O)C=C1		
		P-Coumaroylquinic acid	107	O=C(O)[C@]1(O)C[C@@H](O)[C@@H](O)[C@H](OC(/C=C/C2=CC=C(O)C=C2)=O)C1		
		Hydroxycoumarin	108	O=C1C(O)=CC2=CC=CC=C2O1		
		Isochlorogenic acid b	109	O=C(O)[C@]1(O)C[C@@H](O)[C@@H](OC(/C=C/C2=CC=C(O)C(O)=C2)=O)[C@H](OC(/C=C/C3=CC=C(O)C(O)=C3)=O)C1		
		Quercetin rhamnoside	110	C[C@H]1[C@H](O)[C@@H](O)[C@@H](O)[C@H](OC2=C(C3=CC=C(O)C(O)=C3)OC4=CC(O)=CC(O)=C4C2=O)O1		
		Isochlorogenic acid C	111	O=C(O)[C@@]1(O)C[C@@H](O)[C@H](OC(/C=C/C2=CC=C(O)C(O)=C2)=O)[C@H](OC(/C=C/C3=CC=C(O)C(O)=C3)=O)C1		
		Hexanal	112	CCCCCC=O	Eo	Bensliman in morocco [5]
		A-pinene	113	CC1=CC[C@@H]2C[C@H]1C2(C)C		
		Camphene	114	CC(C1=C)(C)[C@@H]2CC[C@H]1C2		
		B-Pinene	115	CC([C@@H]1C2)(C)[C@@H]2CCC1=C		
		Myrcene	116	CC(C)=CCCC(C=C)=C		
		P-cymene	117	CC1=CC=C(C(C)C)C=C1		
		Limonene	118	CC1=CC[C@H](C(C)=C)CC1		
		Linalool	119	CC(C)=CCC[C@@](C=C)(O)C		
		Nonanol	120	CCCCCCCCCO		
		Estragole	121	COC1=CC=C(CC=C)C=C1		
		A-terpineol	122	CC1=CC[C@H](C(C)(O)C)CC1		
		E-anethole	123	C/C=C/C1=CC=C(OC)C=C1		
		Thymol	124	CC1=CC=C(C(C)C)C(O)=C1		
		Bornyl acetate	125	CC(O[C@H]1C[C@@H]2CC[C@@]1(C)C2(C)C)=O		

		Carvacrol	126	CC1=CC=C(C(C)C)C=C1O		
		Nerylacetate	127	CC(C)=CCC/C(C)=C\COC(C)=O		
		Geranylacetone	128	CC(C)=CCC/C(C)=C/CCC(C)=O		
		(E)-β-Fernesene	129	CC(C)=CCC/C(C)=C/C/C=C(C=C)\C		
		B-humulene	130	C/C1=C\CC(C)(C)/C=C/CC(CCC1)=C		
		Alpha-Muurolene	131	CC1=C[C@@H]2[C@@H](C(C)=CC[C@H]2C(C)C)CC1		
		Germacrene D	132	C/C1=C\CCC(/C=C/[C@H](C(C)C)CC1)=C		
		B-Bisabolene	133	CC1=CC[C@H](C(CCC=C(C)C)=C)CC1		
		Cubebol	134	C[C@@H]1CC[C@@H](C(C)C)[C@H]2[C@]13[C@@H]2[C@@](C)(O)CC3		
		Cis-3-Hexenylbenzoate	135	CC/C=C\CCOC(C1=CC=CC=C1)=O		
		Spathulenol	136	C[C@]1(O)CC[C@@H]2[C@@H]1[C@H]3[C@@H](CCC2=C)C3(C)C		
		Caryophylene oxide	137	CC1(C)C[C@@H]2[C@@H]1CC[C@]3(C)[C@H](CCC2=C)O3		
		4(14)-Salvialene-1-one	138	CC([C@@H]1CC[C@]2(C)[C@H]1CC(CCC2=O)=C)C		
		Caryophylla-4(14), 8(15)-dien-5-ol	139	CC1(C)C[C@@H]2[C@@H]1CCC([C@@H](O)CCC2=C)=C		
		Vulgarone-B	140	CC1=CC([C@@H]2[C@@H]3[C@H]1[C@@]2(C)CCCC3(C)C)=O		
		A-cadinol	141	CC1=C[C@H]2[C@H](C(C)C)CC[C@@](C)(O)[C@@H]2CC1		
		A-bisabolol	142	CC1=CC[C@H]([C@@](CCC=C(C)C)(O)C)CC1		
		Phytone	143	CC(CCC[C@H](CCC[C@H](CCCC(C)=O)C)C)C		
		Caffeic acid	6	OC1=CC(/C=C/C(O)=O)=CC=C1O	Cold maceration ethanol	Leaves, empty capitulas, seeds, and roots from the Timahdite [6]
		Hydroxytyrosol	144	OCCC1=CC=C(O)C(O)=C1		
		L-arginine	279	O=C(O)[C@@H](N)CCCN=C(N)N		
		Gallic acid	1	OC(C1=CC(O)=C(O)C(O)=C1)=O		
		Pellitorine	103	CCCCC/C=C/C=C/C(NCC(C)C)=O		
		Catechin	2	OC1=CC2=C(C[C@@H](O)[C@H](C3=CC=C(O)C(O)=C3)O2)C(O)=C1		
		Vanillic acid	5	OC1=C(OC)C=C(C(O)=O)C=C1		
		Chlorogenic acid	4	OC1=CC=C(C=C1O)/C=C/C(O[C@H]2[C@H](O)[C@@H](O)C[C@@](O)(C(O)=O)C2)=O		
		Coumarin	146	O=C1C=CC2=CC=CC=C2O1		

		Cinnamic acid	93	O=C(O)/C=C/C1=CC=CC=C1		
		P-coumaric acid	11	OC1=CC=C(/C=C/C(O)=O)C=C1		
		Trans ferulic acid	15	COC1=C(O)C=CC(/C=C/C(O)=O)=C1		
		Ferulic acid	15	COC1=C(O)C=CC(/C=C/C(O)=O)=C1		
		Oleuropein	148	C/C=C1[C@H](CC(OCCC2=CC=C(O)C(O)=C2)=O)C(C(OC)=O)=CO[C@H]\1O[C@H]3[C@H](O)[C@@H](O)[C@H](O)[C@@H](CO)O3		
		Naringin	16	OC1=CC=C(C=C1)[C@H]2CC(C3=C(O2)C=C(C=C3O)O[C@@H]4O[C@H](CO)[C@@H](O)[C@H](O)[C@H]4O[C@@H]5[C@@H](O)[C@H](O)[C@@H](O)[C@H](C)O5)=O		
		Quercetin	7	OC1=CC(O)=C(C(C(O)=C(C2=CC=C(O)C(O)=C2)O3)=O)C3=C1		
		Geraniol	149	CC(C)=CCC/C(C)=C/CO		
		Hesperetin	24	OC1=CC2=C(C(C[C@H](C3=CC=C(OC)C(O)=C3)O2)=O)C(O)=C1		
		Deca-2E,4E-dienoic acid N-Me IBA	154	CCCCC/C=C/C=C/C(N(C)CC(C)C)=O		
		Anacyclin	155	CCCC#CC#CCC/C=C/C=C/C(NCC(C)C)=O		
	Artemisia mesatlantica L.	Santolinatriene	156	CC(C)=C[C@H](C(C)=C)C=C	Eo	The leaves and flowering tops collected in the central middle atlas mountain zone [7]
		Tricyclene	157	CC1(C)[C@@H]2C[C@H]3[C@]1(C)[C@H]3C2		
		A-Thujene	158	CC1=CC[C@]2(C(C)C)[C@H]1C2		
		A-Pinene	113	CC1=CC[C@@H]2C[C@H]1C2(C)C		
		Camphene	114	CC(C1=C)(C)[C@@H]2CC[C@H]1C2		
		Sabinene	159	CC([C@]1([C@H]2C1)CCC2=C)C		
		B-pinene	115	CC([C@@H]1C2)(C)[C@@H]2CCC1=C		
		1-decene	160	CCCCCCCCC=C		
		Myrcene	116	CC(C)=CCCC(C=C)=C		
		Yomogi alcohol	161	CC(C=C)(/C=C/C(C)(O)C)C		
		A-Terpinene	162	CC1=CC=C(C(C)C)CC1		
		P-Cymene	117	CC1=CC=C(C(C)C)C=C1		
		O-Cymene	164	CC1=C(C(C)C)C=CC=C1		
		1,8-cineole	165	CC1(C)[C@@H]2CC[C@](CC2)(C)O1		
		Santolina alcohol	166	CC(C)=C[C@H](C(C)(O)C)C=C		
		B -(E)-Ocimene	167	CC(C)=CC/C=C(C=C)\C		

		Bergamal	168	C[C@H](C=O)CCC=C(C)C		
		Artemisia ketone	169	CC(C)=CC(C(C)(C=C)C)=O		
		Cis-Sabinene hydrate	170	CC([C@@]12CC[C@](C)(O)[C@@H]1C2)C		
		Cis-Thujone	171	C[C@H]1[C@@H]2C[C@@]2(C(C)C)CC1=O		
		Trans-Thujone	226	C[C@H]1[C@H]2C[C@@]2(C(C)C)CC1=O		
		Neo-Isopulegol	173	C[C@H]1CC[C@H](C(C)=C)[C@@H](O)C1		
		Chrysanthenone	174	CC([C@@H]1C2(C)C)=CC[C@@H]2C1=O		
		Allo-Ocimene	175	C/C=C(/C=C/C=C(C)C)C		
		Trans-Pinocarveol	176	CC1(C)[C@@H]2C[C@H]1C([C@@H](O)C2)=C		
		Trans-ρ-Menth-2-en-1-ol	177	C[C@]1(O)C=C[C@H](C(C)C)CC1		
		Trans-Sabinol	178	CC([C@@]1([C@@H]2C1)C[C@@H](O)C2=C)C		
		Camphor	179	CC1(C)[C@@H](C2)CC[C@@]1(C)C2=O		
		Pinocarvone	180	CC1(C)[C@@H](C2)C[C@H]1C(C2=O)=C		
		Borneol	181	CC1(C)[C@@H]2CC[C@@]1(C)[C@H](O)C2		
		4-hexen-1-ol,5-methyl-2-(1-methylethenyl) Lavandulol	182	CC(C)=CC[C@H](C(C)=C)CO		
		Terpinen-4-ol	183	CC1=CC[C@@](C(C)C)(O)CC1		
		Thuj-3-en-10-al	184	CC([C@@]12CC=C(C=O)[C@@H]1C2)C		
		Prenylangelate	367	C/C(C([O-])=O)=C(CC=C(C)C)/C		
		Myrtenal	185	CC1(C)[C@@H]2CC=C(C=O)[C@H]1C2		
		Myrtenol	186	CC1(C)[C@@H]2CC=C(CO)[C@H]1C2		
		Γ-Terpineol	187	CC(C)=C1CCC(C)(O)CC1		
		Trans-Piperitol	188	CC1=C[C@H](O)[C@@H](C(C)C)CC1		
		Endo-Fenchyl acetate	189	CC(O[C@H]1C(C)(C)[C@@H]2CC[C@@]1(C)C2)=O		
		Piperitone	190	CC1=CC([C@H](C(C)C)CC1)=O		
		Cis-Chrysanthenylacetate	191	CC1=CC[C@@H]2[C@@H](OC(C)=O)[C@H]1C2(C)C		
		Isobornylacetate	192	CCCC(=O)O[C@H]1C[C@@H]2CC[C@]1(C2(C)C)C		
		Exo-Arbozol	330	OC[C@@H]1[C@](C2)(C(C)=C)C(C)=C(C)[C@@H]2C1		
		Sesquicineole<7-epi-1,2-dehydro->	337	CC(C)=CCC[C@]1(C)O[C@]2(C)C=C[C@H]1CC2		
		Germacrene D	132	C/C1=C\CCC(/C=C/[C@H](C(C)C)CC1)=C		

		Spathulenol	136	C[C@]1(O)CC[C@@H]2[C@@H]1[C@H]3[C@@H](CCC2=C)C3(C)C		
		Globulol	193	C[C@@H]1CC[C@@H]2[C@@H]1[C@H]3[C@@H](CC[C@]2(O)C)C3(C)C		
		Viridiflorol	194	C[C@@H]1CC[C@H]2[C@@H]1[C@H]3[C@@H](CC[C@@]2(O)C)C3(C)C		
		Eremoligenol	195	C[C@H]1CCC=C2[C@]1(C)C[C@H](C(C)(O)C)CC2		
		A-Cadinol	141	CC1=C[C@H]2[C@H](C(C)C)CC[C@@](C)(O)[C@@H]2CC1		
		Botrydiol	229	C[C@@H]1C[C@@H]([C@@H]2[C@]([C@H]1CO)([C@@](CC2(C)C)(C)CO)O)OC(=O)C		
	Artemisia vulgaris L.	Camphene	114	CC(C1=C)(C)[C@@H]2CC[C@H]1C2	Eo	Leaves from different locations in morocco [8]
		3,3,6-trimethyl-1,4-heptadien-6-ol	196	CC(C=C)(/C=C/C(C)(O)C)C		
		O-cymol	164	CC1=C(C(C)C)C=CC=C1		
		Cineole	165	CC1(C)[C@@H]2CC[C@](CC2)(C)O1		
		4-carene	215	CC1CC2C(C2(C)C)C=C1		
		Butyric acid, 3-hexenyl ester	199	CCCC(OCC/C=C\CC)=O		
		2,7-dimetyl-2,6-octadien-4-ol	200	CC(C)=CC[C@H](O)C=C(C)C		
		B,-thujone	172	C[C@H]1[C@H]2C[C@@]2(C(C)C)CC1=O		
		A,-thujone	201	C[C@@H]1[C@H]2C[C@@]2(C(C)C)CC1=O		
		Fenchene	202	CC1(C)[C@@H](C2)CC[C@H]1C2=C		
		Trans-Pinocarveol	176	CC1(C)[C@@H]2C[C@H]1C([C@@H](O)C2)=C		
		Camphor	179	CC1(C)[C@@H](C2)CC[C@@]1(C)C2=O		
		3-pinanone	203	C[C@@H]1[C@@H]2C[C@H](CC1=O)C2(C)C		
		Santolina triene	156	CC(C)=C[C@H](C(C)=C)C=C		
		Borneol	181	CC1(C)[C@@H]2CC[C@@]1(C)[C@H](O)C2		
		A-lonol	310	CC1=CCCC(C1/C=C/C(C)O)(C)C		
		4-terpineol	183	CC1=CC[C@@](C(C)C)(O)CC1		
		Myrtenol	186	CC1(C)[C@@H]2CC=C(CO)[C@H]1C2		
		Isopinocampheol	239	CC1C2CC(C2(C)C)CC1O		
		6-(1-butenyl)-1,4-cycloheptadiene	208	CC/C=C\[C@H]1CC=CCC=C1		
		A,-limonene diepoxide	246	CC12CCC(CC1O2)C3(CO3)C		

		Piperitone	190	CC1=CC([C@H](C(C)C)CC1)=O		
		Chrysanthenyl acetate	242	CC1=CCC2C(C1C2(C)C)OC(=O)C		
		3-carene, 2-(acetylmethyl)	213	CC1=CCC2C(C1CC(=O)C)C2(C)C		
		Bornyl acetate	125	CC(O[C@H]1C[C@@H]2CC[C@@]1(C)C2(C)C)=O		
		6-hexadecen-4-yne	212	CCCCCCCCC/C=C/C#CCCC		
		Copaene	335	CC1=CC[C@H]2[C@H]3[C@@H]1[C@@]2(CC[C@H]3C(C)C)C		
		Caryophyllene	214	C/C1=C/CCC([C@H]2CC(C)(C)[C@@H]2CC1)=C		
		Gamma-muurolene	217	CC1=C[C@@H]2[C@H](CC1)C(=C)CC[C@H]2C(C)C		
		Germacrene D	132	C/C1=C\CCC(/C=C/[C@H](C(C)C)CC1)=C		
		Gamma-Elemene	216	CC(C)=C1CC[C@@](C)(C=C)[C@H](C(C)=C)C1		
		A-muurolene	131	CC1=C[C@@H]2[C@@H](C(C)=CC[C@H]2C(C)C)CC1		
		Delta-cadinene	346	CC1=C[C@H]2[C@@H](CCC(=C2CC1)C)C(C)C		
		Spathulenol	136	C[C@]1(O)CC[C@@H]2[C@@H]1[C@H]3[C@@H](CCC2=C)C3(C)C		
		Carotol	219	CC1=CC[C@@]2(C)CC[C@H](C(C)C)[C@@]2(O)CC1		
	Artemisia ifranensis L.	A-Pinene	113	CC1=CC[C@@H]2C[C@H]1C2(C)C	Essential oils	Leaves from Timahdite
		B-Pinene	115	CC([C@@H]1C2)(C)[C@@H]2CCC1=C		
		Cymene	117	CC1=CC=C(C(C)C)C=C1		
		Cineole	165	CC1(C)[C@@H]2CC[C@](CC2)(C)O1		
		Terpinene	183	CC1=CC[C@@](C(C)C)(O)CC1		
		Sabinene hydtrate	97	CC(C)[C@]12CC[C@@](C1C2)(C)O		
		Linalool oxide	299	C[C@@]1(C=C)CC[C@H](C(C)(O)C)O1		
		Trans-Sabinenehydrate	365	CC(C)[C@]12CC[C@@](C1C2)(C)O		
		Thujone trans	227	C[C@H]1[C@H]2C[C@@]2(C(C)C)CC1=O		
		Thujone cis	172	C[C@H]1[C@H]2C[C@@]2(C(C)C)CC1=O		
		Fenchol	228	CC([C@@H]1O)(C)[C@@H]2CC[C@@]1(C)C2		
		Thujanol <iso-3>	204	CC1C2CC2(CC1OC(=O)C)C(C)C		
		Sabinol	178	CC([C@@]1([C@@H]2C1)C[C@@H](O)C2=C)C		
		Terpinen-4-ol	183	CC1=CC[C@@](C(C)C)(O)CC1		
		Terpineol	122	CC1=CC[C@H](C(C)(O)C)CC1		
		Myrtenol	186	CC1(C)[C@@H]2CC=C(CO)[C@H]1C2		

		Dihydrocarveol< iso>	348	C[C@H]1CC[C@H](C[C@@H]1O)C(=C)C		
		Fragranol	356	CC(=C)[C@H]1CC[C@]1(C)CCO		
		Dihydrocarveol	235	C[C@H]1CC[C@H](C(C)=C)C[C@@H]1O		
		Sabinyl acetate	234	CC(C)C12CC1C(=C)C(C2)OC(=O)C		
		Dihydrocarveol acetate	237	C[C@H]1CC[C@H](C(C)=C)C[C@@H]1OC(C)=O		
		Caryophylene<E>	214	C/C1=C/CCC([C@H]2CC(C)(C)[C@@H]2CC1)=C		
		Germacrene-D	240	C/C1=C\CCC(/C=C/[C@H](C(C)C)CC1)=C		
		Ionone<methyl-y>	366	CCC(=O)/C=C/C1C(=C)CCCC1(C)C		
		Cubebol	134	C[C@@H]1CC[C@@H](C(C)C)[C@H]2[C@]13[C@@H]2[C@@](C)(O)CC3		
		Italicene epoxide	369	CC1CCC2C13CCC4(C(C3C2(C)C)O4)C		
		Patchouli alcool	243	C[C@H]1CC[C@]2(O)[C@]3(C)[C@H]1C[C@@H](CC3)C2(C)C		
		Palustrol	236	CC1CCC2(C1C3C(C3(C)C)CCC2C)O		
		Dendrolasin	245	CC(C)=CCC/C(C)=C/CCC1=COC=C1		
		Spathulenol	136	C[C@]1(O)CC[C@@H]2[C@@H]1[C@H]3[C@@H](CCC2=C)C3(C)C		
		Caryophyllene oxide	137	CC1(C)C[C@@H]2[C@@H]1CC[C@]3(C)[C@H](CCC2=C)O3		
		Salvial-4(14)-en-1-one	247	CC([C@@H]1CC[C@]2(C)[C@H]1CC(CCC2=O)=C)C		
		Guaiol	248	CC([C@@H]1CC[C@@]([C@H]2C1)(C)CCCC2=C)=C		
		Cedrol	249	C[C@@H]1CC[C@@H](C2(C)C)[C@]13CC[C@@](C)(O)[C@@H]2C3		
		Eremoligenol	195	C[C@H]1CCC=C2[C@]1(C)C[C@H](C(C)(O)C)CC2		
		Caryophylla-4(12),8(13)-dien-5β-ol	250	CC1(C)C[C@H]2[C@H]1CCC([C@@H](O)CCC2=C)=C		
		Hinesol	251	C[C@H]1CCC=C(C)[C@]12CC[C@@H](C(C)(O)C)C2		
		Khusimol	252	CC1(C)[C@@H]2CC[C@@]3([C@@H](CO)CC[C@@H]3C1=C)C2		
		A-Cadinol	141	CC1=C[C@H]2[C@H](C(C)C)CC[C@@](C)(O)[C@@H]2CC1		
		Patchouli alcohol	243	C[C@H]1CC[C@]2(O)[C@]3(C)[C@H]1C[C@@H](CC3)C2(C)C		
		Bisabolol oxide B	253	CC1=CC[C@H]([C@]2(C)CC[C@H](C(C)(O)C)O2)CC1		
		Isobornyl isobutanoate	254	CC(C(O[C@H]1C[C@@H](C2(C)C)CC[C@]12C)=O)C		
		Valeranone	255	CC([C@H]1CC[C@@]([C@]2(C)C1)(C)CCCC2=O)C		
		Guaia-3,10(14)-dien-11-ol	256	CC1=CC[C@H]2[C@H]1C[C@@H](C(C)(O)C)CCC2=C		

		Bisabolone oxide A	257	CC1=CC[C@@H]([C@]2(C)CCC(C(C)(C)O2)=O)CC1		
		Curcumen-12-ol	258	CC1=CCC([C@@H](CC/C=C(CO)/C)C)=CC1		
		Cedr-8(15)-en-9- α -ol acetate	259	C[C@@H]1CC[C@H]2C(C)([C@H]3C[C@@]12C[C@H](OC(C)=O)C3=C)C		
		Bisabolol oxide-A	260	CC1=CC[C@@H]([C@]2(C)CC[C@H](O)C(C)(C)O2)CC1		
		Lanceol	261	CC1=CC[C@@H](C(CC/C=C(CO)\C)=C)CC1		
		Cedryl acetate	262	C[C@@H]1CC[C@@H](C2(C)C)[C@]13CC[C@@](C)(OC(C)=O)[C@@H]2C3		
		Cedren-13-ol acetate	263	C=C1CC[C@]2([C@@H]3CC[C@H]2C)C[C@@H]1[C@]3(COC(C)=O)C		
		Chenopodiol	264	CC1=CCC[C@]2(C)[C@@H]1[C@H](O)[C@@H](C(C)(O)C)CC2		
Fabaceae	*Vicia faba* L.	4-hydroxyphenyl acetic acid	265	O=C(O)CC1=CC=C(O)C=C1	Olive mill waste water	Olive mill in Marrakech [9]
		Gallic acid	1	OC(C1=CC(O)=C(O)C(O)=C1)=O		
		Veratric acid	266	COC1=CC=C(C(O)=O)C=C1OC		
		Paracoumaric acid	11	OC1=CC=C(/C=C/C(O)=O)C=C1		
		Caffeic acid	6	OC1=CC(/C=C/C(O)=O)=CC=C1O		
		Oleuropein	148	C/C=C1[C@H](CC(OCCC2=CC=C(O)C(O)=C2)=O)C(C(OC)=O)=CO[C@H]\1O[C@H]3[C@H](O)[C@@H](O)[C@H](O)[C@@H](CO)O3		
Lamiaceae	*Ajuga iva* L.	Abutasterone	267	C[C@@]1([C@@]2(O)CC[C@@H]1[C@]([C@H](O)C[C@H](O)C(C)(O)C)(O)C)CC[C@H]3C2=CC([C@H]4[C@]3(C)C[C@H](O)[C@H](O)C4)=O	Ethanolic extract	Aerial parts (Morocco) Taroudant [10]
		Cyasterone	268	C[C@H]1[C@H](C[C@@H](O)[C@@]([C@H]2CC[C@]3(O)[C@]2(C)CC[C@H]4C3=CC([C@H]5[C@]4(C)C[C@H](O)[C@H](O)C5)=O)(O)C)[C@@H](C)OC1=O		
		24-hydroxycyasterone	269	O=C1[C@@H]2C[C@H](O)[C@@H](O)C[C@]2(C)[C@@H]3C([C@@](CC[C@@H]4[C@](O)(C)[C@H](O)C[C@@]5(O)[C@H](C)C(O[C@@H]5C)=O)(O)[C@]4(C)CC3)=C1		
		22-Dehydrocyasterone 2-glucoside	270	O=C1[C@@H]2C[C@H](O)[C@@H](O)C[C@]2(C)[C@@H]3C([C@@](CC[C@@H]4[C@](O)(C)C(C[C@H]5[C@H](C)C(O[C@@H]5C)=O)=O)(O)[C@]4(C)CC3)=C1		

		20-hydroxyecdysone	271	C[C@H]([C@H](O)C[C@H](O)C(C)(O)C)[C@H]1CC[C@]2(O)[C@]1(C)CC[C@@H]3C2=CC([C@H]4[C@]3(C)C[C@H](O)[C@H](O)C4)=O		
		Makisterone A	272	C[C@@H](C(C)(O)C)C[C@@H](O)[C@@]([C@H]1CC[C@]2(O)[C@]1(C)CC[C@H]3C2=CC([C@H]4[C@]3(C)C[C@H](O)[C@H](O)C4)=O)(O)C		
		Ponasterone A	273	CC(CC[C@@H](O)[C@@]([C@H]1CC[C@]2(O)[C@]1(C)CC[C@H]3C2=CC([C@H]4[C@]3(C)C[C@H](O)[C@H](O)C4)=O)(O)C)C		
		Sidisterone	274	O=C1C=C[C@@](O1)(C)[C@@H]2[C@]3(C)[C@](CC2)(O)C4=CC([C@@H]5C[C@@H](O)[C@@H](O)C[C@]5(C)[C@H]4CC3)=O		
		Galacturonic acid	275	O[C@@H]1[C@@H](O)[C@@H](O)[C@@H](C(O)=O)O[C@H]1O	Decoction extracts	Leaves from the masmouda region of morocco 17
		Myricetin	276	OC1=CC(C2=C(O)C(C3=C(O)C=C(O)C=C3O2)=O)=CC(O)=C1O		
		Gallic acid	1	OC(C1=CC(O)=C(O)C(O)=C1)=O		
		Ascorbic acid	277	O=C1C(O)=C(O)[C@H](O1)[C@@H](O)CO		
		Mucic acid	278	O=C(O)[C@H](O)[C@@H](O)[C@@H](O)[C@H](O)C(O)=O		
		Arginine	279	O=C(O)[C@@H](N)CCCN=C(N)N		
		Vanillin	280	COC1=CC(C=O)=CC=C1O		
		Quinic acid	281	O=C(O)[C@]1(O)C[C@@H](O)[C@@H](O)[C@H](O)C1		
		Cinnamic acid	93	O=C(O)/C=C/C1=CC=CC=C1		
		Rhamnetin	282	COC1=CC(O)=C2C(OC(C3=CC=C(O)C(O)=C3)=C(O)C2=O)=C1		
		Catechin-7-O-glucoside	283	OC1=CC(O[C@H]2[C@H](O)[C@@H](O)[C@H](O)[C@@H](CO)O2)=CC3=C1C[C@H](O)[C@@H](C4=CC=C(O)C(O)=C4)O3		
		Catechin	2	OC1=CC2=C(C[C@@H](O)[C@H](C3=CC=C(O)C(O)=C3)O2)C(O)=C1		
		Harpagid	284	C[C@]1(O)C[C@@H](O)[C@@]2(O)[C@@H]1[C@H](O[C@H]3[C@H](O)[C@@H](O)[C@H](O)[C@@H](CO)O3)OC=C2		
		Ajugasterone D	285	C[C@@]1([C@@]2(O)CC[C@H]1[C@]([C@@H]3CCC(C)(C)O3)(O)C)CC[C@H]4C2=CC([C@@]5(O)[C@]4(C)C[C@H](O)[C@H](O)C5)=O		
		Coumarin	146	O=C1C=CC2=CC=CC=C2O1		
		Ferulic acid 4-O-glucoside	286	COC1=CC(/C=C/C(O)=O)=CC=C1O[C@H]2[C@H](O)[C@@H](O)[C@H](O)[C@@H](CO)O2		

		Coumaric acid	11	OC1=CC=C(/C=C/C(O)=O)C=C1		
		Epigallocatechin gallate	288	OC1=CC(O)=CC2=C1C[C@@H](OC(C3=CC(O)=C(O)C(O)=C3)=O)[C@@H](C4=CC(O)=C(O)C(O)=C4)O2		
		8-O-acetyl-harpagid	289	CC(O[C@@]1(C)C[C@@H](O)[C@@]2(O)[C@@H]1[C@H](O[C@H]3[C@H](O)[C@@H](O)[C@H](O)[C@@H](CO)O3)OC=C2)=O		
		Ferulic acid	15	COC1=C(O)C=CC(/C=C/C(O)=O)=C1		
		Cholesterol	290	C[C@@H]([C@H]1CC[C@@H]2[C@]1(C)CC[C@H]3[C@H]2CC=C4[C@]3(C)CC[C@H](O)C4)CCCC(C)C		
		Cyasterone	268	C[C@H]1[C@H](C[C@@H](O)[C@@]([C@H]2CC[C@]3(O)[C@]2(C)CC[C@H]4C3=CC([C@H]5[C@]4(C)C[C@H](O)[C@H](O)C5)=O)(O)C)[C@@H](C)OC1=O		
		Resveratrol 3-glucoside	291	OC[C@@H]1[C@@H](O)[C@H](O)[C@@H](O)[C@@H](O1)OC2=CC(O)=CC(/C=C/C3=CC=C(O)C=C3)=C2		
		Apigenin-7-(2-O-apiosylglucoside)	292	O=C1C(C(OC(C2=CC=C(O)C=C2)=C1)=C3)=C(O)C=C3O[C@H]4[C@@H]([C@@H](O)[C@H](O)[C@@H](CO)O4)O[C@H]5[C@@H](O)[C@](CO)(O)CO5		
		Apigenin 7-O-(6″-malonyl-apiosyl-glucoside)	293	O=C(C1=C(O)C=C(O[C@H]2[C@H](O)[C@@H](O)[C@H](O)[C@@H](CO[C@H]3[C@H](O)[C@H](O)[C@@H](COC(CC(O)=O)=O)O3)O2)C=C1O4)C=C4C5=CC=C(O)C=C5		
		Apigenin	294	O=C(C1=C(O)C=C(O)C=C1O2)C=C2C3=CC=C(O)C=C3		
		Quercetin-3-O-pentosyl-pentoside	295	O=C1C(O[C@H]2[C@@H]([C@@H](O)[C@H](O)CO2)O[C@H]3[C@H](O)[C@@H](O)[C@H](O)CO3)=C(C4=CC=C(O)C(O)=C4)OC5=CC(O)=CC(O)=C51		
		Quercetin	7	OC1=CC(O)=C(C(C(O)=C(C2=CC=C(O)C(O)=C2)O3)=O)C3=C1		
		Kaempferide	296	COC1=CC=C(C2=C(O)C(C3=C(O)C=C(O)C=C3O2)=O)C=C1		
		Luteolin	22	O=C1C2=C(O)C=C(O)C=C2OC(C3=CC(O)=C(O)C=C3)=C1		
		Trans-p-coumaric acid	11	O=C(O)/C=C/C1=CC=C(O)C=C1		

		Vanillic acid glucoside	298	COC1=CC(C(O)=O)=CC=C1O[C@H]2[C@H](O)[C@@H](O)[C@H](O)[C@@H](CO)O2		
	Lavandula angustifolia L.	Cis-linalool oxide	299	C[C@@]1(C=C)CC[C@H](C(C)(O)C)O1	Eo	Collected in the rural municipality of talsint, situated in figuig-Morocco [11]
		Camphene	114	CC(C1=C)(C)[C@@H]2CC[C@H]1C2		
		B-Pinene	115	CC([C@@H]1C2)(C)[C@@H]2CCC1=C		
		Trans-linalool oxide	300	C[C@@]1(C=C)CC[C@@H](C(C)(O)C)O1		
		P-cymene	117	CC1=CC=C(C(C)C)C=C1		
		1,8-cineole	165	CC1(C)[C@@H]2CC[C@](CC2)(C)O1		
		Camphor	179	CC1(C)[C@@H](C2)CC[C@@]1(C)C2=O		
		Terpinen-4-ol	183	CC1=CC[C@@](C(C)C)(O)CC1		
		Caryophyllene	214	C/C1=C/CCC([C@H]2CC(C)(C)[C@@H]2CC1)=C		
		Linalool	119	CC(C)=CCC[C@@](C=C)(O)C		
		Borneol	181	CC1(C)[C@@H]2CC[C@@]1(C)[C@H](O)C2		
		Linalyl acetate	301	CC(C)=CCC[C@@](C=C)(OC(C)=O)C		
		(+)-Epi-bicyclosesquiphellandrene	302	C[C@H]1CC[C@@H](C(C)C)C2=CC(CC[C@H]12)=C		
	Lavandula maroccana L.	Furfural	303	O=CC1=CC=CO1	Eo	Aerial parts from ijoukak region [12]
		5-hydroxymethyl furfural	304	O=CC1=CC=C(CO)O1		
		1-Octen-3-ol	305	CCCCC[C@H](O)C=C		
		1,8-cineole	165	CC1(C)[C@@H]2CC[C@](CC2)(C)O1		
		A -Terpinene	162	CC1=CC=C(C(C)C)CC1		
		Linalool	119	CC(C)=CCC[C@@](C=C)(O)C		
		Terpinen-4-ol	183	CC1=CC[C@@](C(C)C)(O)CC1		
		P-Cymen-8-ol	308	CC1=CC=C(C(C)(O)C)C=C1		
		A-Terpineol	122	CC1=CC[C@H](C(C)(O)C)CC1		
		2.3-dihydrobenzofuran	309	C1(C=CC=C2)=C2CCO1		
		Isopiperitone	190	CC(CC[C@H]1C(C)C)=CC1=O		
		Thymol	124	CC1=CC=C(C(C)C)C(O)=C1		

		Carvacrol	126	CC1=CC=C(C(C)C)C=C1O		
		2-Methoxy-4-vinylphenol	311	COC1=CC(C=C)=CC=C1O		
		A -Terpinyl acetate	312	CC1=CC[C@H](C(C)(OC(C)=O)C)CC1		
		Methyleugenol	313	COC1=CC=C(CC=C)C=C1OC		
		B-Caryophyllene	314	C/C1=C/CCC([C@H]2CC(C)(C)[C@@H]2CC1)=C		
		A-Farnesene	297	CC(=CCC/C(=C/C/C=C(/C)\C=C)/C)C		
		Spathulenol	136	C[C@]1(O)CC[C@@H]2[C@@H]1[C@H]3[C@@H](CCC2=C)C3(C)C		
		Caryophyllene oxide	137	CC1(C)C[C@@H]2[C@@H]1CC[C@]3(C)[C@H](CCC2=C)O3		
	L,pedunculata L. *L,angustifolia* L.	Eucalyptol	165	CC1(C)[C@@H]2CC[C@](CC2)(C)O1	Eo	Aerial parts khenifra, ifrane and volubilis respectively [13]
		Fenchone	316	CC(C1=O)(C)[C@@H]2CC[C@@]1(C)C2		
		2,4-Di-tertbutylphenol	317	CC(C)(C1=CC=C(O)C(C(C)(C)C)=C1)C		
	Mentha suaveolens L.	Quinic acid	281	O=C(O)[C@]1(O)C[C@@H](O)[C@@H](O)[C@H](O)C1	Methanol extract	The aerial parts from middle Atlas of Morocco (Ifrane) [14]
		THDBCHMCA: 1,2,6,7-tetrahydroxy-5H-dibenzo- [a,d]cycloheptene-5-methyl-11-carboxylic acid	318	C[C@@H]1C(C(O)=C(O)C=C2)=C2C=C3C4=C1C=CC(O)=C4OC3=O		
		Luteolin-dihexoside	319	O=C1C=C(OC2=CC(O[C@@H](O3)[C@@H](O)[C@H](O)[C@@H](O)[C@@H]3CO)=CC(O[C@@H](O4)[C@@H](O)[C@H](O)[C@@H](O)[C@@H]4CO)=C12)C5=CC(O)=C(O)C=C5		
		Luteolin-hexoside	320	O=C1C=C(C2=CC=C(O)C(O)=C2)OC3=CC(O)=CC(O[C@@H]4[C@H]([C@@H]([C@H]([C@H](CO)O4)O)O)O)=C13		
		Luteolin-glucuronide	321	O=C1C=C(OC2=CC(O)=CC(O[C@@H](O3)[C@H](O)[C@@H](O)[C@H](O)[C@H]3C(O)=O)=C12)C4=CC(O)=C(O)C=C4		
		Apigenin-dihexoside	322	O=C1C=C(C2=CC=C(O)C=C2)OC3=CC(O[C@@H]4[C@H]([C@@H]([C@H]([C@H](CO)O4)O)O)O)=CC(O[C@@H]5[C@H]([C@@H]([C@H]([C@H](CO)O5)O)O)O)=C13		
		Apigenin-glucuronide	323	O=C1C=C(C2=CC=C(O)C=C2)OC3=CC(O)=CC(O[C@@H]4[C@@H]([C@H]([C@@H]([C@@H](C(O)=O)O4)O)O)O)=C13		

		Salvianolic acid B	324	O=C(O)[C@H](OC(/C=C/C1=CC=C(O)C2=C1[C@H](C(O[C@@H](C(O)=O)CC3=CC=C(O)C(O)=C3)=O)[C@@H](C4=CC=C(O)C(O)=C4)O2)=O)CC5=CC=C(O)C(O)=C5		
		Rosmarinic acid	325	O=C(O)[C@H](OC(/C=C/C1=CC=C(O)C(O)=C1)=O)CC2=CC=C(O)C(O)=C2		
		Salvianolic acid J		C1=CC(=C(C=C1CC(C(=O)O)OC(=O)/C=C/C2=C3C(C(OC3=C(C=C2)O)C4=CC(=C(C=C4)O)O)C(=O)OC(CC5=CC(=C(C=C5)O)O)C(=O)O)O)O		
		Dihydroxy-tetramethoxyflavone	327	O=C1C(OC)=C(C2=CC=CC=C2O)OC3=C(O)C(OC)=C(OC)C(OC)=C13		
		Salvianolic acid A	326	O=C(O)[C@H](OC(/C=C/C1=CC=C(O)C(O)=C1/C=C/C2=CC=C(O)C(O)=C2)=O)CC3=CC=C(O)C(O)=C3		
		Jaceosidin	329	COC1=CC(C2=CC(C3=C(O)C(OC)=C(O)C=C3O2)=O)=CC=C1O		
		Salvianolic acid B/E	378	C1=CC(=C(C=C1C[C@H](C(=O)O)OC(=O)/C=C/C2=C(C(=C(C=C2)O)O)/C(=C\C3=CC(=C(C=C3)O)O)/C(=O)O[C@H](CC4=CC(=C(C=C4)O)O)C(=O)O)O)O		
		4-*O*-caffeoylquinic acid	106	O=C(O)[C@]1(O)C[C@@H](O)[C@@H](OC(/C=C/C2=CC=C(O)C(O)=C2)=O)[C@H](O)C1	Hydromethanolic extract	Leaves and flowers from Taounate [15]
		5-*O*-caffeoylquinic acid	331	O=C(O)[C@@]1(O)C[C@@H](O)[C@H](O)[C@H](OC(/C=C/C2=CC=C(O)C(O)=C2)=O)C1		
	Syzygium aromaticum L.	Eugenol	332	COC1=CC(CC=C)=CC=C1O	Eo	Stems, leaves and flowers in morocco [16]
		Caryophyllene	238	C/C1=C/CCC([C@H]2CC(C)(C)[C@@H]2CC1)=C		
		1,1,4,8-tetramethyl-cis,cis,4,7,10-cycloundecatriene	333	C/C1=C/C/C=C(C)\CCC(C)(C)/C=C\C1		
		Caryophyllene oxide	137	CC1(C)C[C@@H]2[C@@H]1CC[C@]3(C)[C@H](CCC2=C)O3		
		Humulene epoxide II	334	C/C1=C\CC(C)(C)/C=C/C[C@]2(C)[C@@H](CC1)O2		
		Aromandendrene	335	C[C@H]1CC[C@@H]2[C@H]1[C@H]3[C@@H](CCC2=C)C3(C)C		
		2-Pentadecen-4-yne, (Z)-	336	CCCCCCCCCCC#C/C=C\C		

		Δ-Cadinene	346	CC1=C[C@H]2[C@@H](CCC(=C2CC1)C)C(C)C		
		Trans-Calamenene	338	C[C@H]1CC[C@H](C(C)C)C2=C1C=CC(C)=C2		
		1-(3,4-methylenedioxyphenyl)propane-1-ol	339	CCC(C1=CC2=C(C=C1)OCO2)O		
		Estragole	121	COC1=CC=C(CC=C)C=C1		
Pinaceae	*Pinus halepensis* L.	A-Pinene	113	CC1=CC[C@@H]2C[C@H]1C2(C)C	Eo	Needles of aleppo pine tree from the park tazekka, in taza-region fez-meknes (morocco) [17]
		1R-α-Pinene	209	CC1=CC[C@@H]2C[C@H]1C2(C)C		
		Sabinene	159	CC([C@]1([C@H]2C1)CCC2=C)C		
		4(10)-thujene	159	CC([C@]1([C@H]2C1)CCC2=C)C		
		B-Pinene	115	CC([C@@H]1C2)(C)[C@@H]2CCC1=C		
		Trans-β-Ocimene	167	CC(C)=CC/C=C(C=C)\C		
		4-Isopropylidene-1- cyclohexene	223	CC(=C)C1CCC=CC1		
		Copaene	341	CC1=CC[C@H]2[C@H]3[C@@H]1[C@@]2(CC[C@H]3C(C)C)C		
		Caryophyllene	214	C/C1=C/CCC([C@H]2CC(C)(C)[C@@H]2CC1)=C		
		A-Bisabolene	342	CC1=CC[C@H](/C(C)=C/CC=C(C)C)CC1		
		A-Humulene	343	C/C1=C\CC(C)(C)/C=C/C/C(C)=C/CC1		
		Isovalerate de β-phenylethy	344	CC(CC(OCCC1=CC=CC=C1)=O)C		
		A-muurolene	131	CC1=C[C@@H]2[C@@H](C(C)=CC[C@H]2C(C)C)CC1		
		A-Cadinene	345	CC1=C[C@@H]2[C@H](C(C)=CC[C@H]2C(C)C)CC1		
		Caryophyllene oxide	137	CC1(C)C[C@@H]2[C@@H]1CC[C@]3(C)[C@H](CCC2=C)O3		
		Guaiol	248	CC([C@@H]1CC[C@@]([C@H]2C1)(C)CCCC2=C)=C		
		Caryophyllene-(I1)	214	C/C1=C/CCC([C@H]2CC(C)(C)[C@@H]2CC1)=C		
		Cembrene	241	C/C/1=C\CC/C(=C/C/C=C(\C=C\C(CC1)C(C)C)/C)/C		
		Cembrene A	347	C/C1=C\CC/C(C)=C/C[C@H](C(C)=C)CC/C(C)=C/CC1		
		Thunbergol	147	C/C/1=C\CCC(/C=C/C(CC/C(=C/CC1)/C)C(C)C)(C)O		
Apiacée	*Coriandrum sativum* L.	A –thujene	158	CC1=CC[C@]2(C(C)C)[C@H]1C2	Oe	Seeds[18]
		Sabinene	340	CC([C@]1([C@H]2C1)CCC2=C)C		
		A-phellandrene	349	CC1=CC[C@@H](C(C)C)C=C1		
		A-terpinene	162	CC1=CC=C(C(C)C)CC1		
		O-Cymene	164	CC1=C(C(C)C)C=CC=C1		
		D-limonene	371	CC1=CCC(CC1)C(=C)C		

		Eucalyptol	165	CC1(C)[C@@H]2CC[C@](CC2)(C)O1		
		(Z)-β-ocimene	350	CC(C)=CC/C=C(C=C)/C		
		Gamma-Terpinene	351	CC1=CCC(C(C)C)=CC1		
		Cis-sabinene	307	CC(C)C12CCC(=C)[C@H]1C2		
		P mentha-1,4 (8) -dene	353	CC1=CCC(CC1)=C(C)C		
		Spiro[4.5]dec-6-ene	354	C1CCC2(CCCC=C2)C1		
		Camphor	179	CC1(C)[C@@H](C2)CC[C@@]1(C)C2=O		
		Pinocarvone	180	CC1(C)[C@@H](C2)C[C@H]1C(C2=O)=C		
		Borneol	181	CC1(C)[C@@H]2CC[C@@]1(C)[C@H](O)C2		
		Terpinen-4-ol	183	CC1=CC[C@@](C(C)C)(O)CC1		
		P Cymen-8-ol	308	CC1=CC=C(C(C)(O)C)C=C1		
		P-menth-1-en-8-ol	122	CC1=CC[C@H](C(C)(O)C)CC1		
		(-)-Myrtenol	101	CC1([C@H]2CC=C([C@@H]1C2)CO)C		
		Decanal	357	CCCCCCCCCC=O		
		(-)-Verbenone	358	CC1=CC([C@H]2C[C@@H]1C2(C)C)=O		
		Citronellol	315	CC(CCC=C(C)C)CCO		
		Nerol	359	CC(C)=CCC/C(C)=C\CO		
		Geranial(citral)	360	CC(C)=CCC/C(C)=C/C=O		
		Cuminaldehyde	361	CC(C1=CC=C(C=O)C=C1)C		
		Geraniol	149	CC(C)=CCC/C(C)=C/CO		
		Methyl citronellate	362	C[C@H](CC(OC)=O)CCC=C(C)C		
		2-decenal, (e)-	363	CCCCCCC/C=C/C=O		
		2-Decen-1-ol	364	CCCCCCC/C=C/CO		
		1-decanol	197	CC1=CC[C@@H]2C[C@H]1C2(C)C		
		Anethole	123	C/C=C/C1=CC=C(OC)C=C1		
		P-Thymol	220	CC1=C(C=CC(=C1)O)C(C)C		
		Undecanal	145	CCCCCCCCCCC=O		
		Myrtenyl acetate	368	CC(OCC1=CC[C@@H]2C[C@H]1C2(C)C)=O		
		Citronellyl acetate	328	CC(CCC=C(C)C)CCOC(=O)C		
		Neryl acetate	370	CC(C)=CCC/C(C)=C\COC(C)=O		
		Dodecanal	372	CCCCCCCCCCCC=O		

		B-Caryophyllene	314	C/C/1=C\CCC(=C)[C@H]2CC([C@@H]2CC1)(C)C		
		Trans-2-dodecenal	373	CCCCCCCCC/C=C/C=O		
		2-decylfuran	374	CCCCCCCCCCC1=CC=CO1		
		Trans-2-Undecen-1-ol	375	CCCCCCCC/C=C/CO		
		Myristicin	376	COC1=CC(CC=C)=CC2=C1OCO2		
		Caryophyllene oxide	137	CC1(C)C[C@@H]2[C@@H]1CC[C@]3(C)[C@H](CCC2=C)O3		
		Gamma-muurolene	217	CC1=C[C@@H]2[C@H](CC1)C(=C)CC[C@H]2C(C)C		
		Phytone	143	CC(CCC[C@H](CCC[C@H](CCCC(C)=O)C)C)C		
	Foeniculum vulgare L.	A-pinene	113	CC1=CC[C@@H]2C[C@H]1C2(C)C	Eo	Seeds in the region of meknes [19]
		Camphene	114	CC(C1=C)(C)[C@@H]2CC[C@H]1C2		
		B-Phellandrene	377	CC([C@H]1CCC(C=C1)=C)C		
		B-pinene	115	CC([C@@H]1C2)(C)[C@@H]2CCC1=C		
		B-myrcene	116	CC(C)=CCCC(C=C)=C		
		A-Phellandrene	349	CC1=CC[C@@H](C(C)C)C=C1		
		P-Cymene	117	CC1=CC=C(C(C)C)C=C1		
		D-limonene	371	CC1=CC[C@@H](CC1)C(=C)C		
		Eucalyptol	165	CC1(C)[C@@H]2CC[C@](CC2)(C)O1		
		Bicyclo[3.1.1]hept-2-ene, 3,6,6-trimethyl-	379	CC1=C[C@@H](C2(C)C)C[C@@H]2C1		
		Γ-Terpinen	351	CC1=CCC(C(C)C)=CC1		
		L-fenchone	232	C[C@@]12CC[C@@H](C1)C(C2=O)(C)C		
		Camphor	179	CC1(C)[C@@H](C2)CC[C@@]1(C)C2=O		
		Terpinene-4-ol	183	CC1=CC[C@@](C(C)C)(O)CC1		
		Estragole	121	COC1=CC=C(CC=C)C=C1		
		Bicyclo[2.2.1]heptan-2-ol, 1,3,3-trimethyl-, acetate, (1S-exo)-	189	CC(O[C@H]1C(C)(C)[C@@H]2CC[C@@]1(C)C2)=O		
		P-Anisaldehyde	383	COC1=CC=C(C=O)C=C1		
		1-Butanone, 2-chloro-3-methyl-1-[4-(1-methylethyl)phenyl]-	102	CC(C)C1=CC=C(C=C1)C(=O)C(C(C)C)Cl		
		4-[(S)-sec-Butyl]anisole	385	COC1=CC=C([C@@H](C)CC)C=C1		
		4-methoxyphenylacetone	386	CC(CC1=CC=C(OC)C=C1)=O		
		Formic acid, 2-isopropylphenyl ester	382	CC(C1=C(OC=O)C=CC=C1)C		

		M-Anisic acid, 4-chlorophenyl ester	381	COC1=CC(C(OC2=CC=C(Cl)C=C2)=O)=CC=C1		
		Myristicin	376	COC1=CC(CC=C)=CC2=C1OCO2		
		1,3-Benzenediamine, N,N,N',N'-tetramethyl	380	CN(C1=CC=CC(N(C)C)=C1)C		
		P-cymene	117	CC1=CC=C(C(C)C)C=C1	Eo	The seeds collected from gourrama, southeastern morocco [20]
		Limonene	118	CC1=CC[C@H](C(C)=C)CC1		
		3-carène	211	CC1=CCC2C(C1)C2(C)C		
		Eucalyptol	165	CC1(C)[C@@H]2CC[C@](CC2)(C)O1		
		ϒ-terpinène	351	CC1=CCC(C(C)C)=CC1		
		Fenchone	316	CC(C1=O)(C)[C@@H]2CC[C@@]1(C)C2		
		Carveol	231	CC1=CCC(CC1O)C(=C)C		
		Camphor	179	CC1(C)[C@@H](C2)CC[C@@]1(C)C2=O		
		4-terpineol	183	CC1=CC[C@@](C(C)C)(O)CC1		
		Estragole	121	COC1=CC=C(CC=C)C=C1		
		P-cumic aldehyde	361	CC(C1=CC=C(C=O)C=C1)C		
		Cis-anethole	244	C/C=C\C1=CC=C(C=C1)OC		
		Para-Anisaldehyde	383	COC1=CC=C(C=O)C=C1		
		Anethole	123	C/C=C/C1=CC=C(OC)C=C1		
		A-Terpinen-7-ol	206	CC1=CC[C@@](C(C)C)(O)CC1		
	Pimpinella anisum L	Camphene	114	CC(C1=C)(C)[C@@H]2CC[C@H]1C2	Eo	Anise seeds was collected from the middle of morocco (meknes) [21]
		Limonene	118	CC1=CC[C@H](C(C)=C)CC1		
		Fenchone	316	CC(C1=O)(C)[C@@H]2CC[C@@]1(C)C2		
		4-allylanisole	121	COC1=CC=C(CC=C)C=C1		
		Anethole	123	C/C=C/C1=CC=C(OC)C=C1		
		Acide linoléique	225	CCCCC/C=C\C/C=C\CCCCCCCC(=O)O		
		A–Pinene	113	CC1=CC[C@@H]2C[C@H]1C2(C)C	Eo	Seeds of green anise from [illegible]
		Camphene	114	CC(C1=C)(C)[C@@H]2CC[C@H]1C2		
		Fenchone	316	CC(C1=O)(C)[C@@H]2CC[C@@]1(C)C2		
		A–Campholene	198	CC1=CC[C@@H](CC=O)C1(C)C		

		Fenchyl acetate	384	CC(=O)OC1C(C2CCC1(C2)C)(C)C		
		Cis-limonene oxide	205	CC([C@@H]1CC[C@](O2)(C)[C@@H]2C1)=C		
		Cis-anethole	244	C/C=C\C1=CC=C(C=C1)OC		
		Isobornyl acetate	207	CC(O[C@H]1C[C@@H]2CC[C@@]1(C)C2(C)C)=O		
		Trans-anethole	123	C/C=C\C1=CC=C(OC)C=C1		
		A-Caryophyllene	343	C/C1=C\CC(C)(C)/C=C/C/C(C)=C/CC2		
		Azulene	218	C12=CC=CC=CC1=CC=C2		
		Ledene	221	C[C@@H]1CCC2=C(C)CC[C@@H](C3(C)C)[C@@H]3[C@H]12		
	Apium graveolens L. *Coriandrum sativum* L. *Petroselinum crispum* L.	Caffeic acid	6	OC1=CC(/C=C/C(O)=O)=CC=C1O	Methanol extract	The celery, coriander, and parsley were collected during the flowering period from the Tinghir region located in the southeast of Morocco [23]
		Chlorogenic acid	4	OC1=CC=C(C=C1O)/C=C/C(O[C@H]2[C@H](O)[C@@H](O)C[C@@](O)(C(O)=O)C2)=O		
		P-Coumaric acid	11	OC1=CC=C(/C=C/C(O)=O)C=C1		
		Ferulic acid	15	COC1=C(O)C=CC(/C=C/C(O)=O)=C1		
		Gallic acid	1	OC(C1=CC(O)=C(O)C(O)=C1)=O		
		Syringic acid	8	COC1=CC(C(O)=O)=CC(OC)=C1O		
		Vanillic acid	5	OC1=C(OC)C=C(C(O)=O)C=C1		
		Luteolin	22	O=C1C2=C(O)C=C(O)C=C2OC(C3=CC(O)=C(O)C=C3)=C1		
		Quercetin	7	OC1=CC(O)=C(C(C(O)=C(C2=CC=C(O)C(O)=C2)O3)=O)C3=C1		
		Rutin	3	OC1=CC(O)=C(C2=C1)C(C(O[C@@H]3[C@@H](O)[C@H](O)[C@@H](O)[C@H](CO[C@H]4O[C@@H](C)[C@H](O)[C@@H](O)[C@@H]4O)O3)=C(O2)C5=CC=C(O)C(O)=C5)=O		
	Apium Graveolens L.	A-Thujene	158	CC1=CC[C@]2(C(C)C)[C@H]1C2	Essential oil	Celery Seed in the area of Marrakech [24]
		Hydroxybutric acid lactone	222	C1CC(=O)OC1		
		B-Pinene	115	CC([C@@H]1C2)(C)[C@@H]2CCC1=C		
		Bèta-myrcene	116	CC(C)=CCCC(C=C)=C		
		D-limonene	371	CC1=CC[C@@H](CC1)C(=C)C		
		6-butyl-1,4-cycloheptadiène	224	CCCCC1CC=CCC=C1		
		Amyl benzene	233	CCCCCC1=CC=CC=C1		
		B-Selinene	248	CC([C@@H]1CC[C@@]([C@H]2C1)(C)CCCC2=C)=C		
		Caryophylene oxide	137	CC1(C)C[C@@H]2[C@@H]1CC[C@]3(C)[C@H](CCC2=C)O3		

		Beta-Selinenol	287	C[C@@]1([C@H]2C[C@H](C(C)(O)C)CC1)CCCC2=C		
		2-chloro-1-(-2,4-dimethylphenyl)-2-methyl-1-propanone	306	CC1=CC=C(C(C(C(C)(Cl)C)=O)C(C)=C1		
		A-Hydroxypropylbenzene	85	CCC(C1=CC=CC=C1)O		
	Carum carvi L.	Ferulic acid	15	COC1=C(O)C=CC(/C=C/C(O)=O)=C1	Maceratio (methenol exctract)	Seedsthe region of taounate (fez, morocco) [25]
		Gallic acid	1	OC(C1=CC(O)=C(O)C(O)=C1)=O		
		Myricetin	276	OC1=CC(C2=C(O)C(C3=C(O)C=C(O)C=C3O2)=O)=CC(O)=C1O		
		Catechin	2	OC1=CC2=C(C[C@@H](O)[C@H](C3=CC=C(O)C(O)=C3)O2)C(O)=C1		
		Caffeic acid	6	OC1=CC(/C=C/C(O)=O)=CC=C1O		
		Quercetin	7	OC1=CC(O)=C(C(C(O)=C(C2=CC=C(O)C(O)=C2)O3)=O)C3=C1		
	Petroselinum crispum	Vanillic acid	5	OC1=C(OC)C=C(C(O)=O)C=C1	Soxhle	The leaves of P. Crispum were collected in
		Luteolin	22	O=C1C2=C(O)C=C(O)C=C2OC(C3=CC(O)=C(O)C=C3)=C1		
		Kaempferol	163	C1=CC(=CC=C1C2=C(C(=O)C3=C(C=C(C=C3O2)O)O)O)O		
		Quercetin	7	OC1=CC(O)=C(C(C(O)=C(C2=CC=C(O)C(O)=C2)O3)=O)C3=C1		
		Apigenin	294	O=C(C1=C(O)C=C(O)C=C1O2)C=C2C3=CC=C(O)C=C3		

Table S2: 2D Structures of Chemical Compounds Extracted from Plants

1.	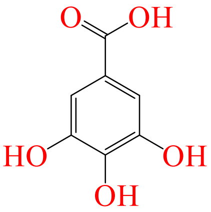
2.	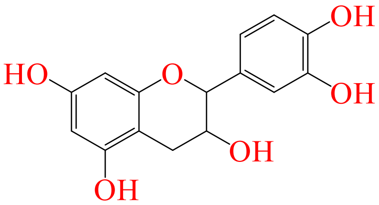
3.	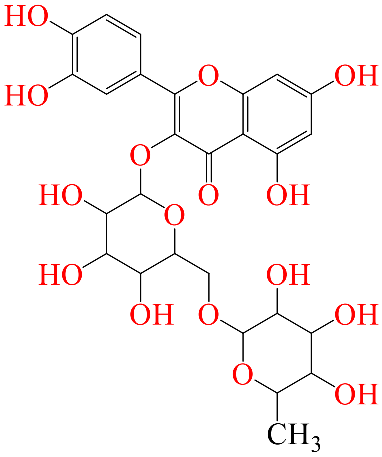
4.	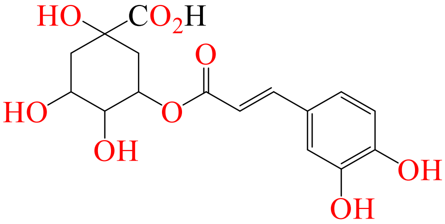
5.	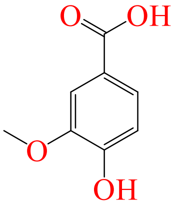
6.	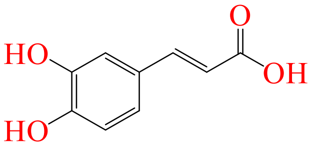
7.	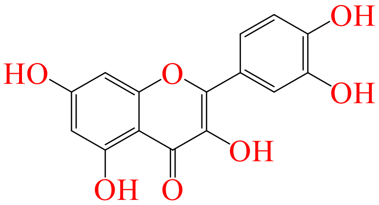

8.	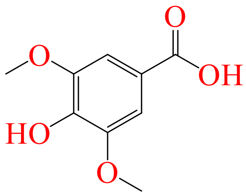
9.	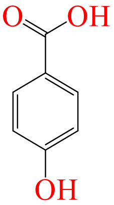
10.	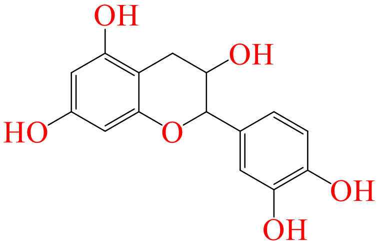
11.	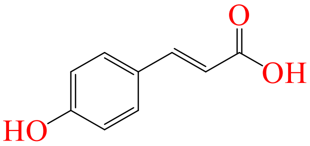
12.	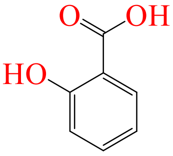
13.	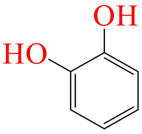
14.	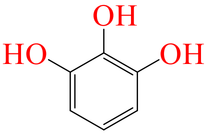
15.	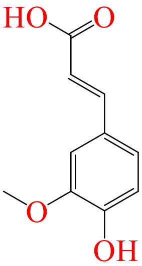

16.	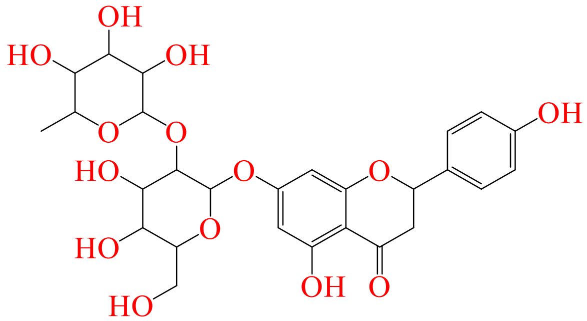
17.	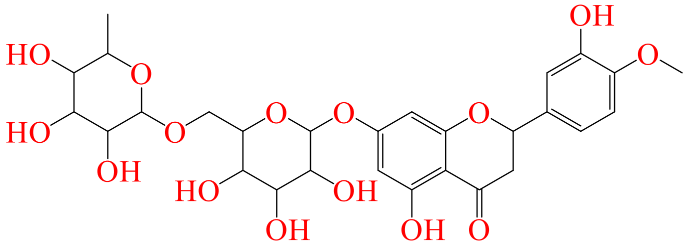
18.	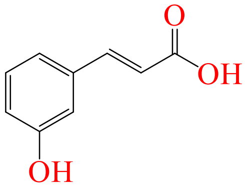
19.	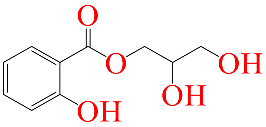
20.	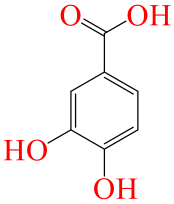
21.	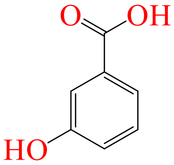
22.	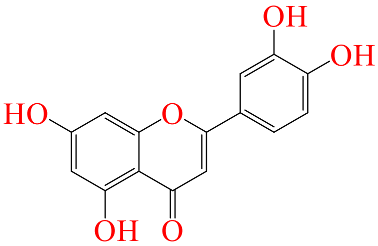
23.	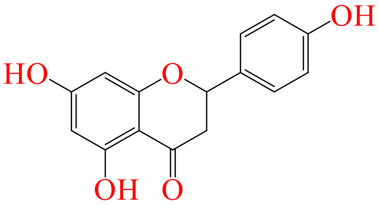

24.	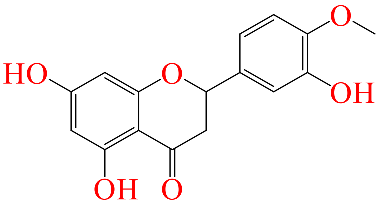
25.	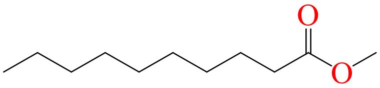
26.	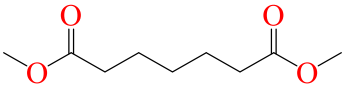
27.	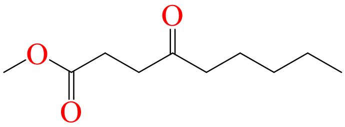
28.	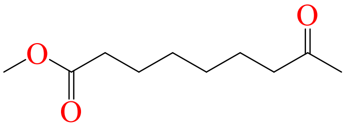
29.	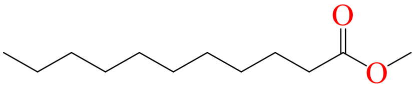
30.	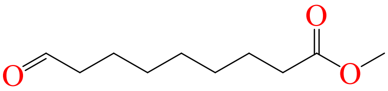
31.	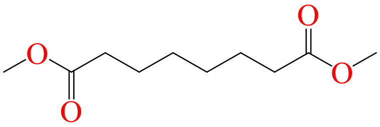
32.	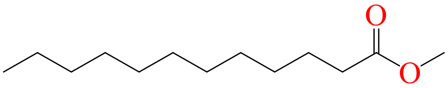
33.	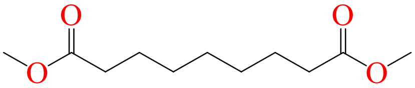
34.	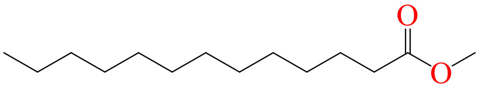
35.	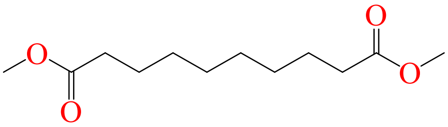
36.	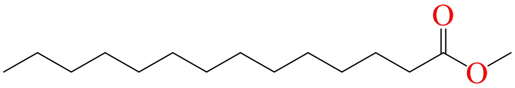
37.	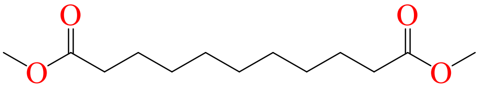
38.	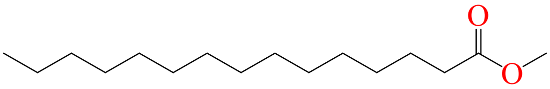

39.	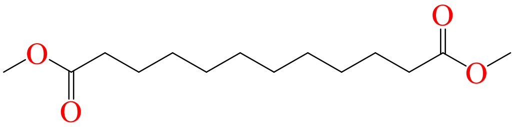
40.	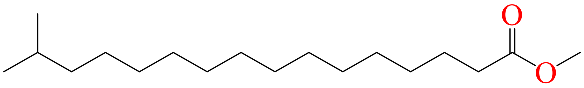
41.	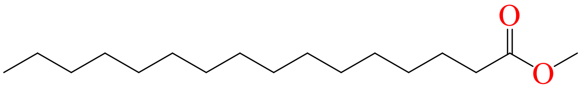
42.	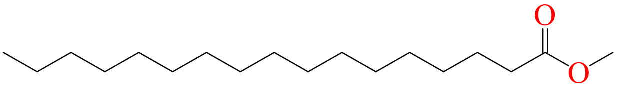
43.	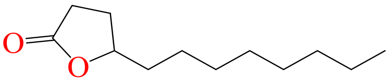
44.	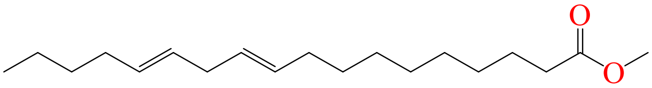
45.	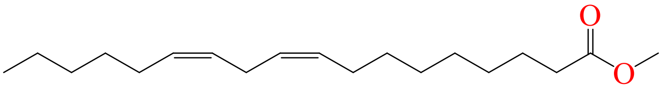
46.	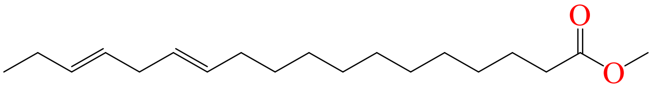
47.	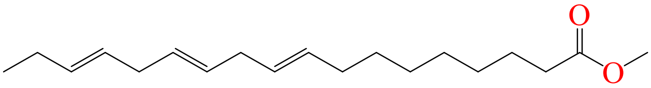
48.	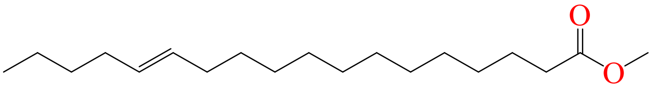
49.	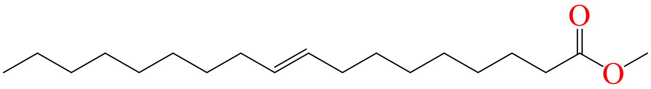
50.	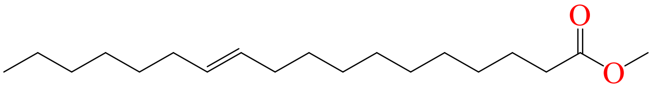
51.	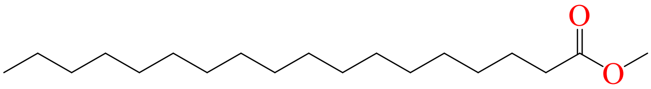
52.	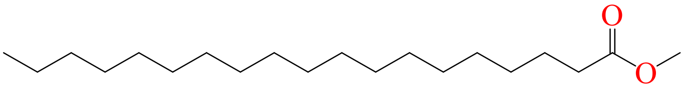
53.	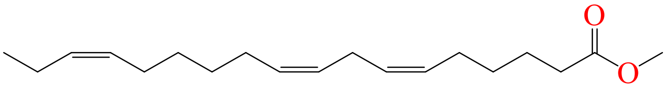
54.	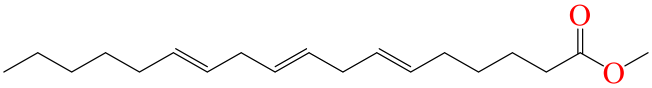
55.	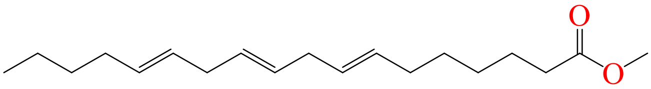
56.	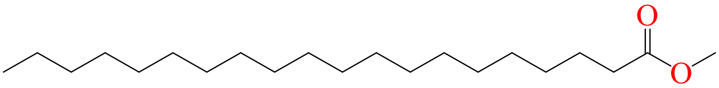

57.	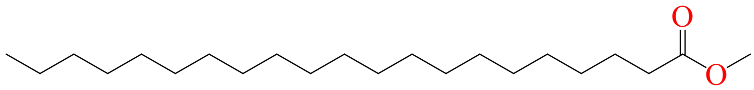
58.	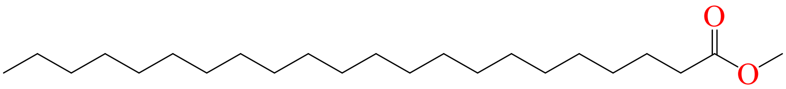
59.	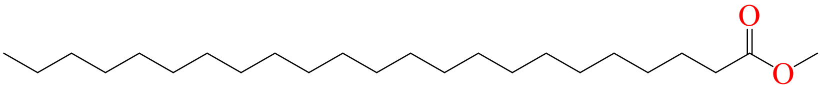
60.	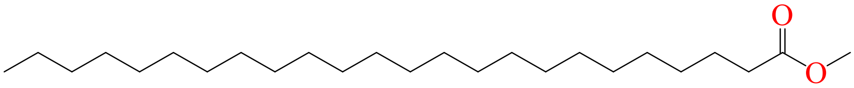
61.	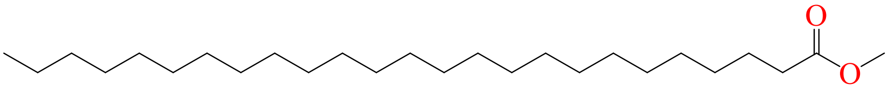
62.	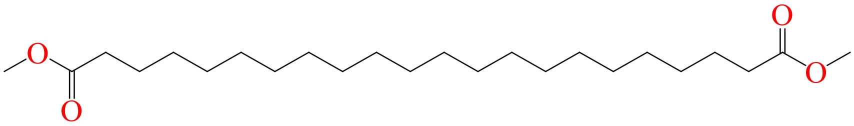
63.	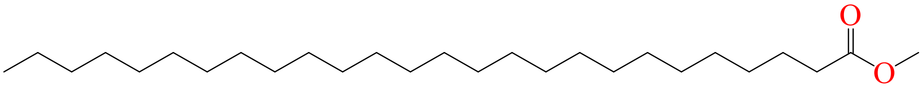
64.	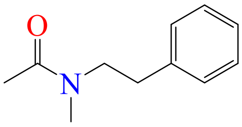
65.	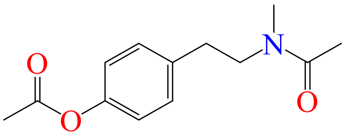
66.	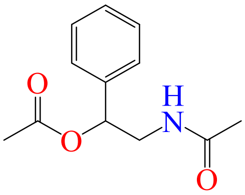
67.	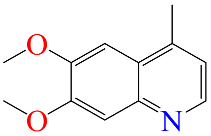
68.	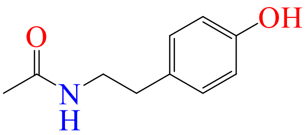
69.	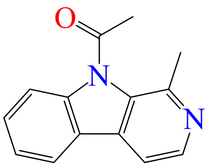
70.	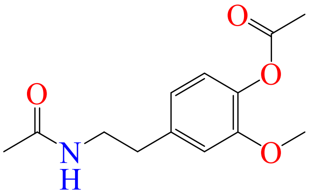

71.	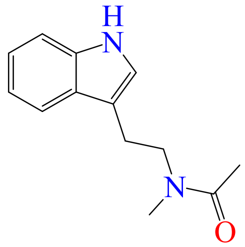
72.	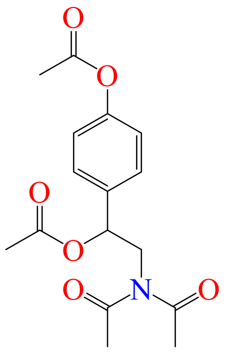
73.	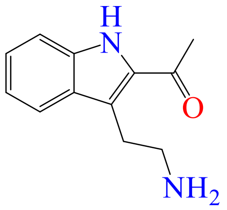
74.	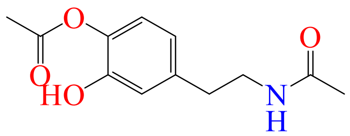
75.	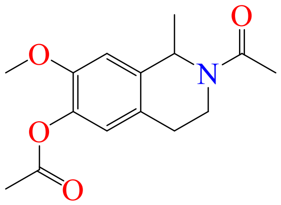
76.	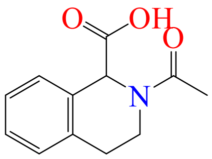
77.	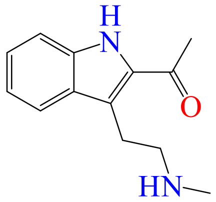
78.	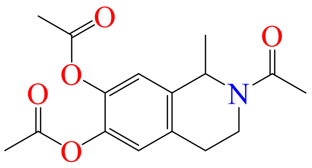

79.	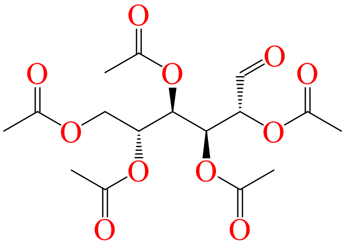
80.	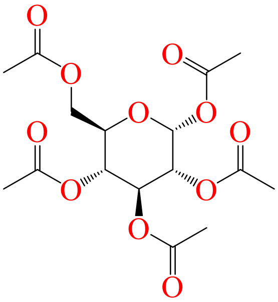
81.	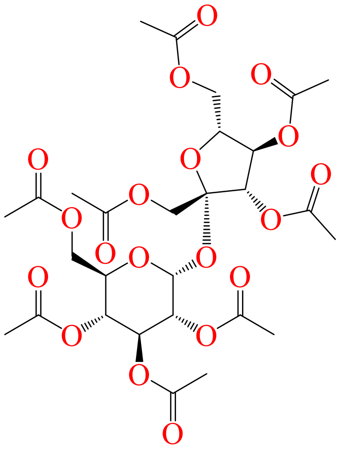
82.	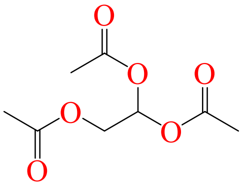
83.	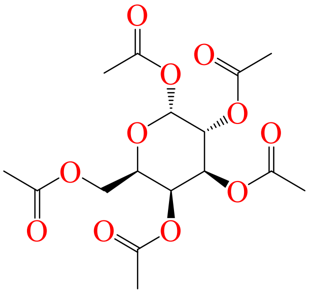
84.	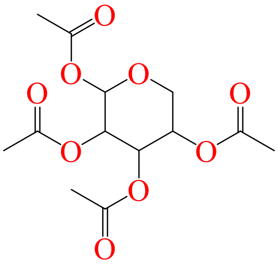

85.	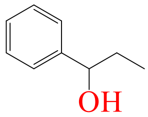
86.	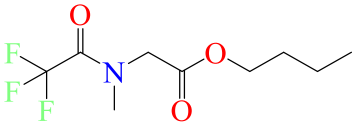
87.	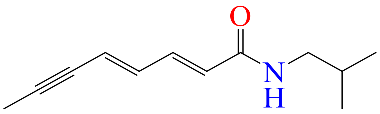
88.	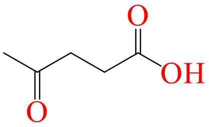
89.	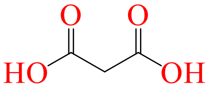
90.	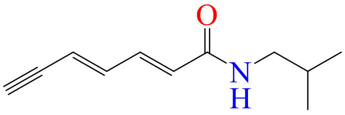
91.	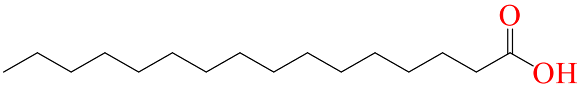
92.	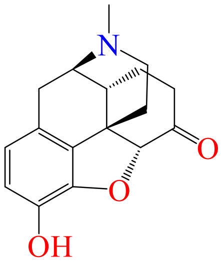
93.	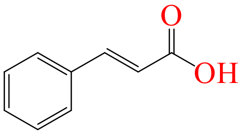
94.	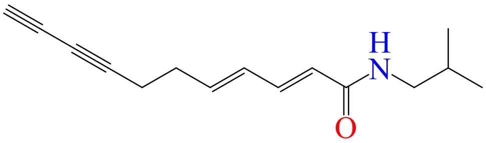
95.	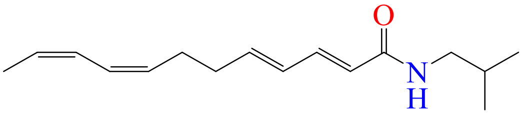
96.	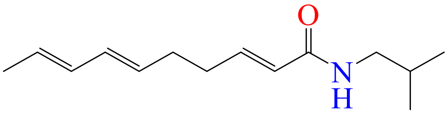
97.	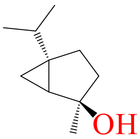

98.	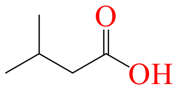
99.	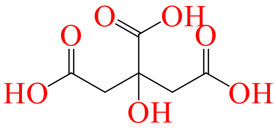
100.	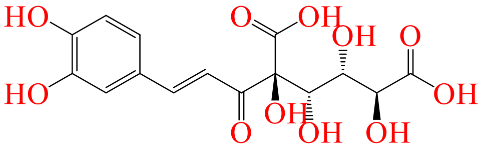
101.	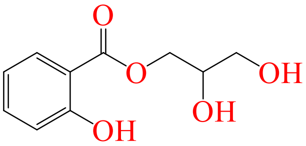
102.	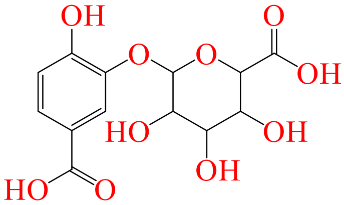
103.	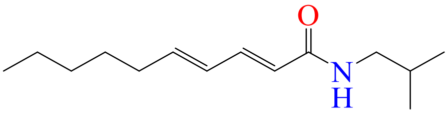
104.	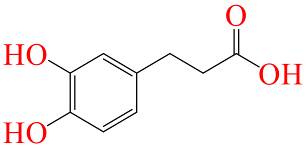
105.	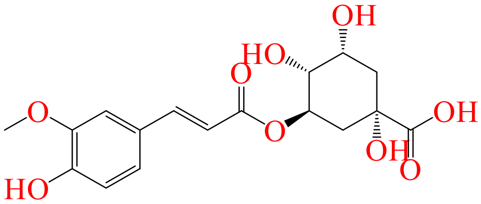
106.	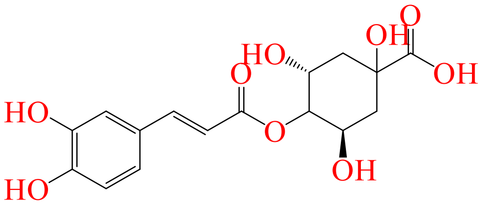
107.	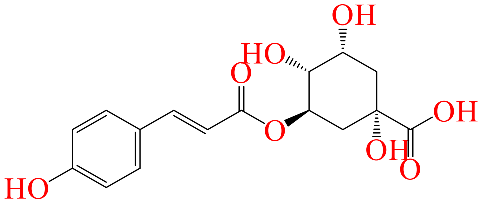
108.	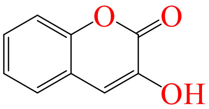

109.	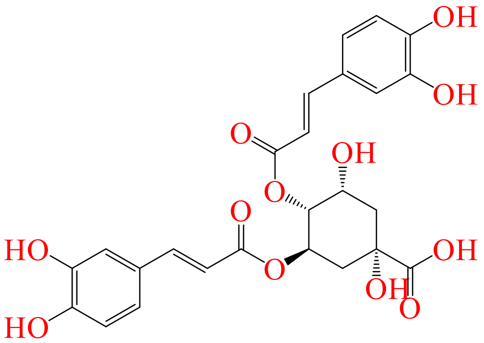
110.	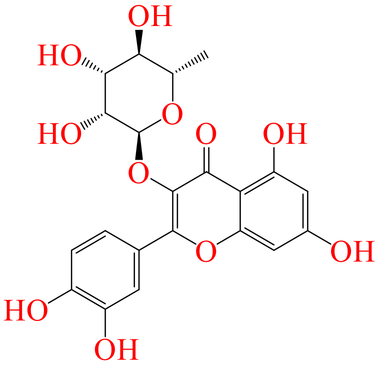
111.	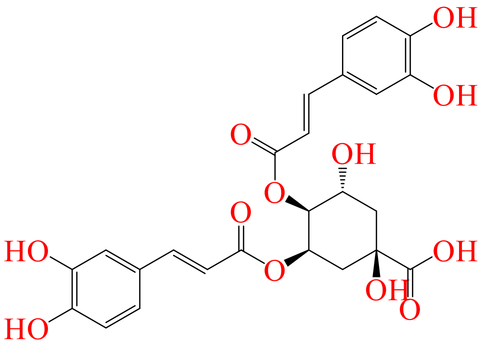
112.	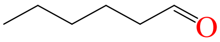
113.	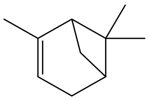
114.	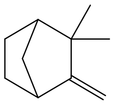
115.	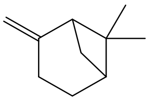
116.	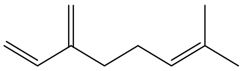
117.	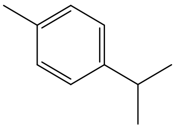
118.	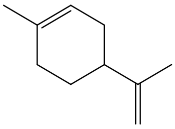
119.	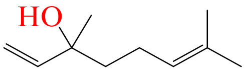

120.	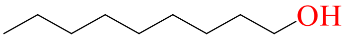
121.	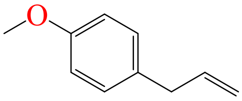
122.	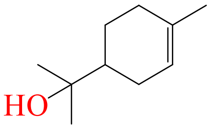
123.	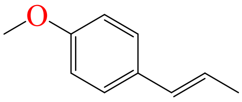
124.	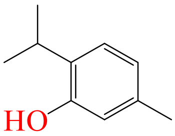
125.	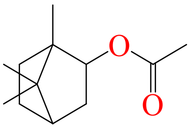
126.	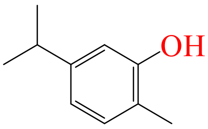
127.	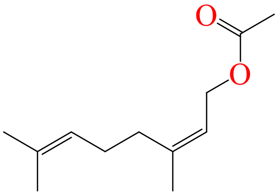
128.	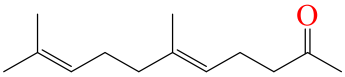
129.	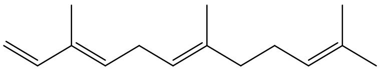
130.	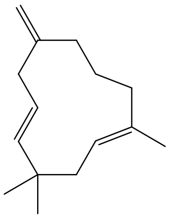
131.	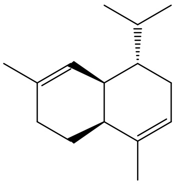
132.	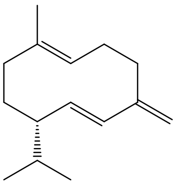

133.	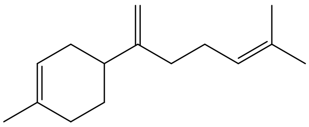
134.	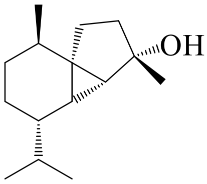
135.	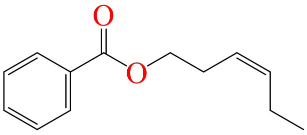
136.	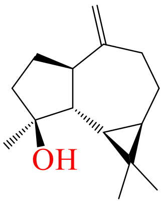
137.	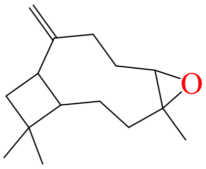
138.	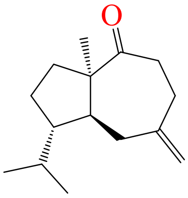
139.	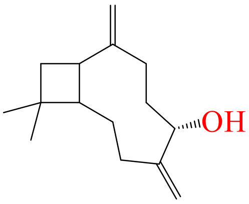
140.	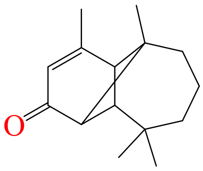
141.	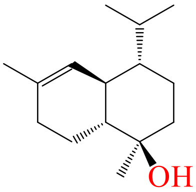
142.	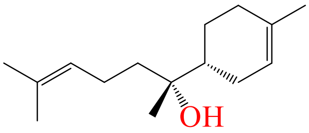
143.	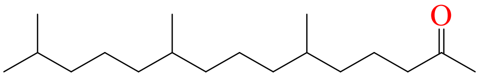

144.	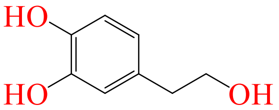
145.	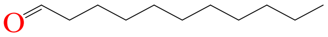
146.	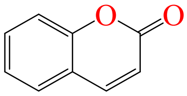
147.	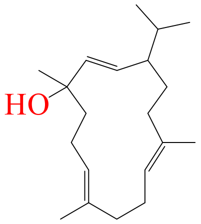
148.	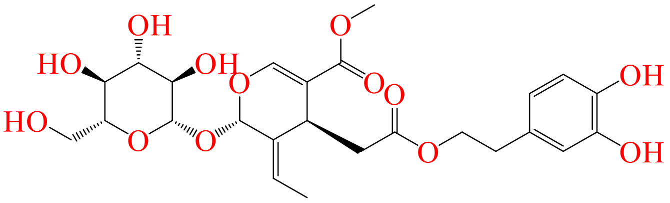
149.	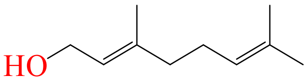
150.	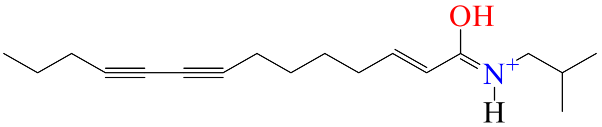
151.	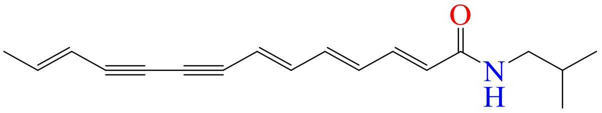
152.	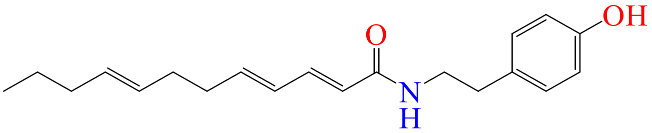
153.	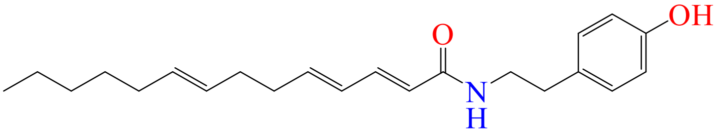
154.	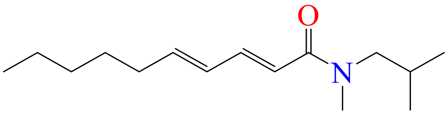
155.	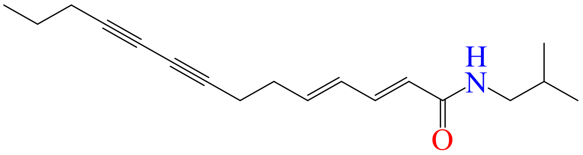
156.	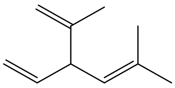
157.	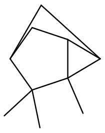

158.	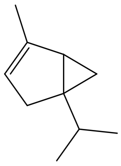
159.	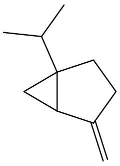
160.	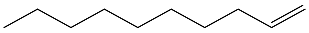
161.	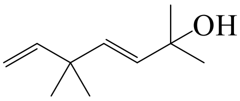
162.	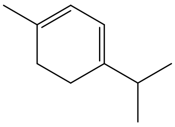
163.	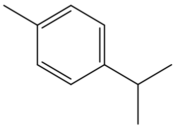
164.	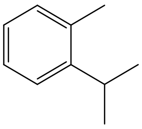
165.	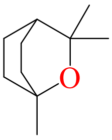
166.	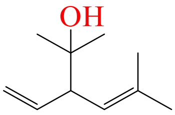
167.	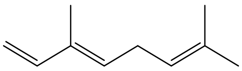
168.	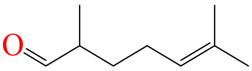
169.	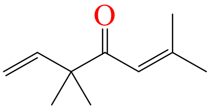
170.	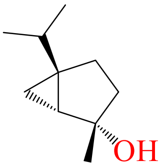
171.	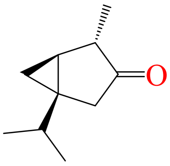

172.	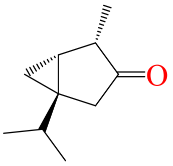
173.	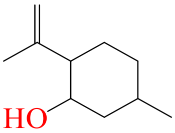
174.	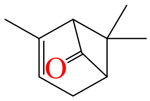
175.	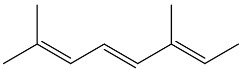
176.	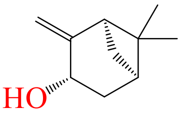
177.	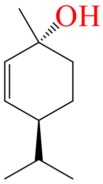
178.	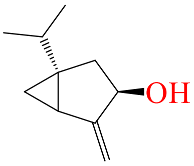
179.	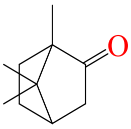
180.	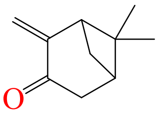
181.	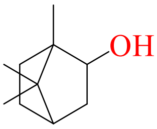
182.	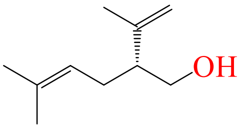
183.	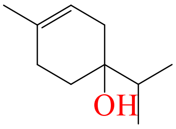
184.	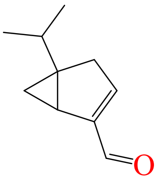

185.	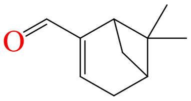
186.	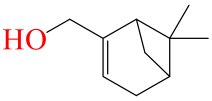
187.	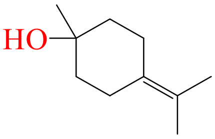
188.	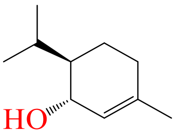
189.	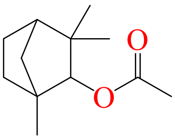
190.	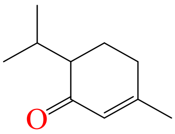
191.	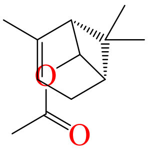
192.	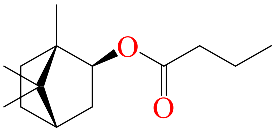
193.	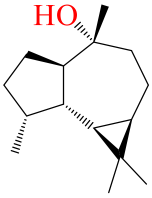
194.	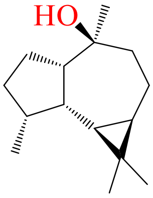
195.	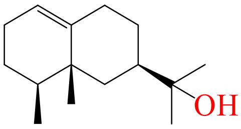
196.	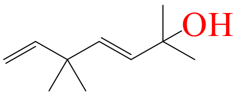
197.	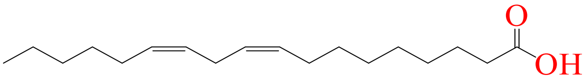

198.	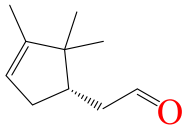
199.	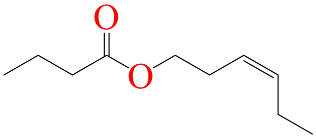
200.	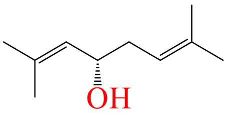
201.	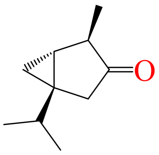
202.	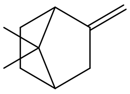
203.	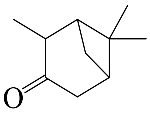
204.	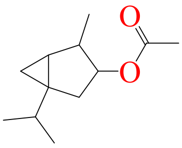
205.	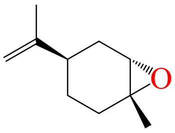
206.	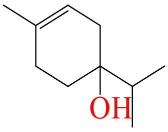
207.	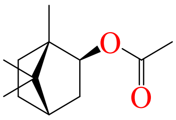
208.	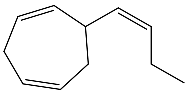
209.	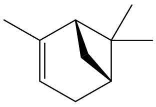
210.	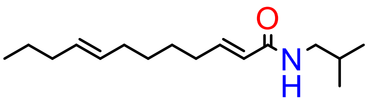
211.	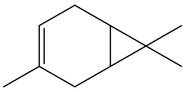
212.	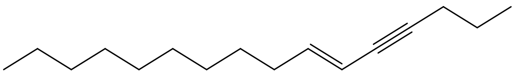

213.	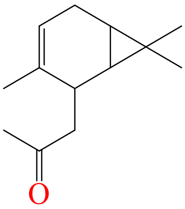
214.	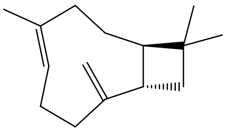
215.	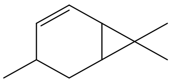
216.	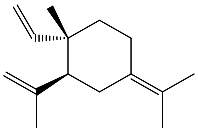
217.	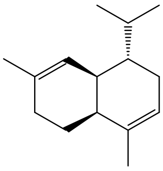
218.	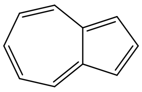
219.	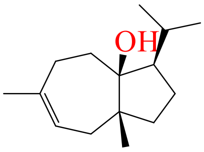
220.	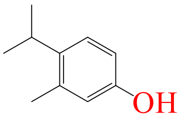
221.	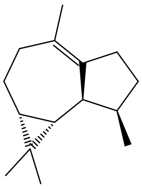
222.	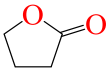
223.	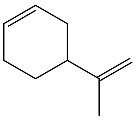
224.	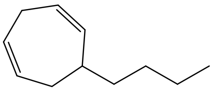
225.	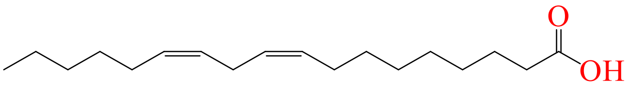
226.	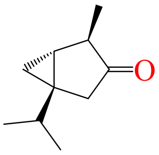

227.	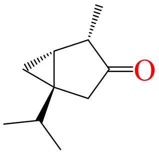
228.	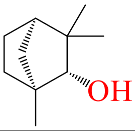
229.	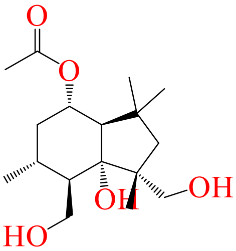
230.	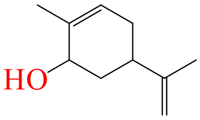
231.	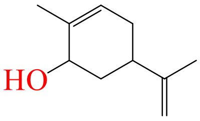
232.	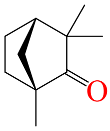
233.	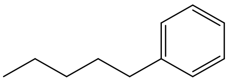
234.	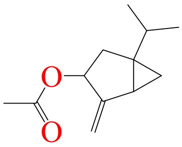
235.	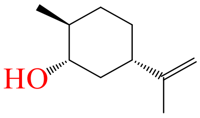
236.	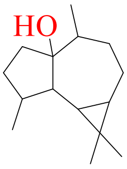
237.	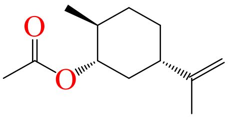
238.	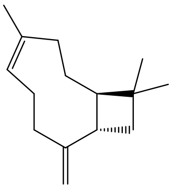

239.	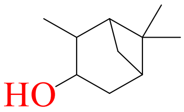
240.	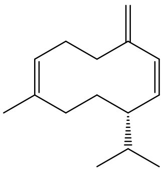
241.	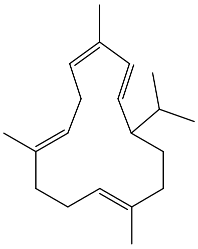
242.	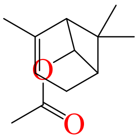
243.	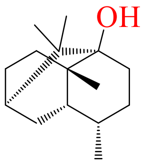
244.	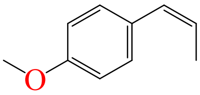
245.	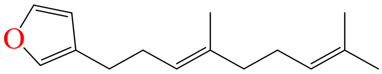
246.	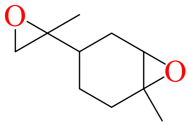
247.	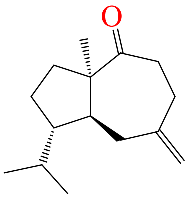
248.	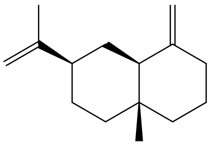
249.	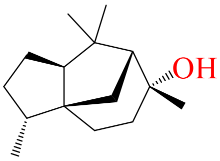

250.	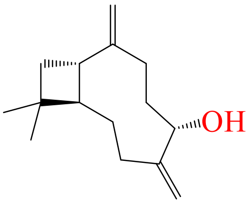
251.	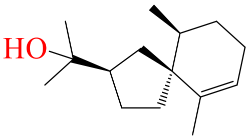
252.	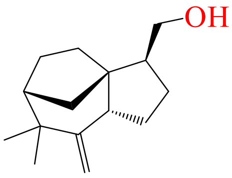
253.	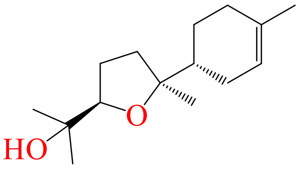
254.	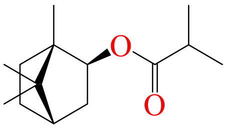
255.	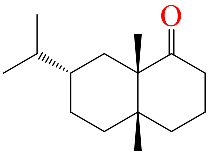
256.	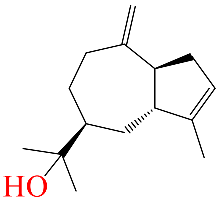
257.	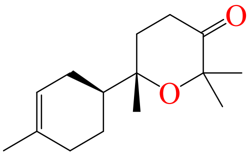
258.	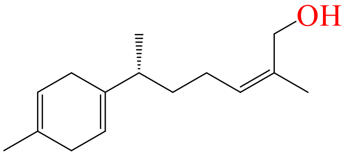
259.	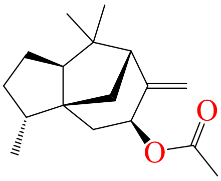

260.	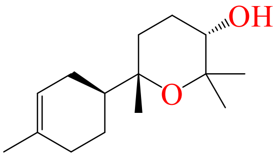
261.	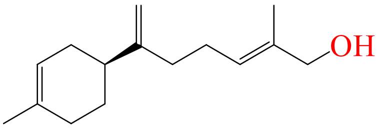
262.	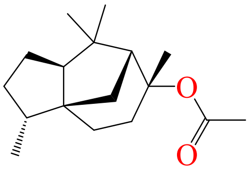
263.	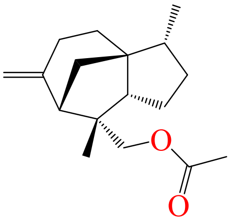
264.	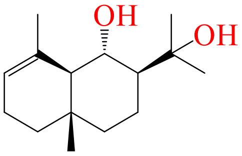
265.	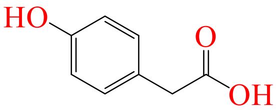
266.	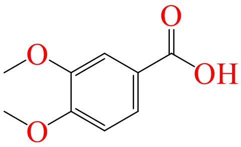
267.	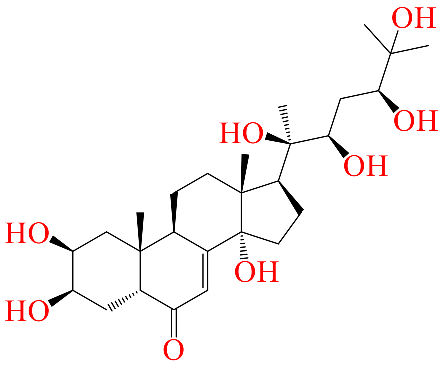
268.	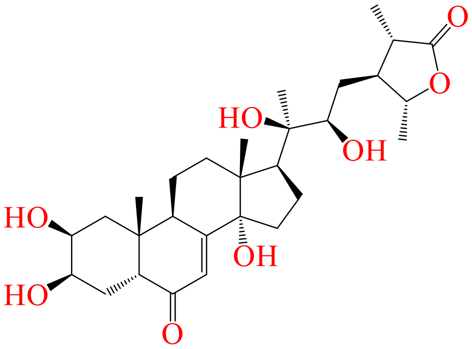

269.	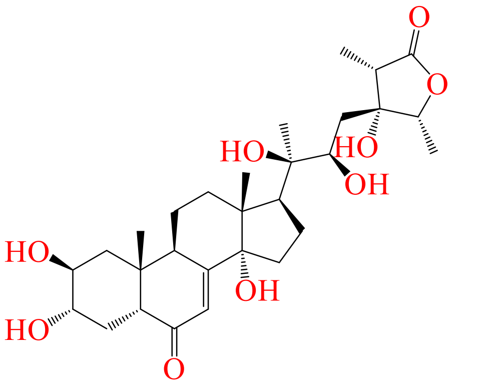
270.	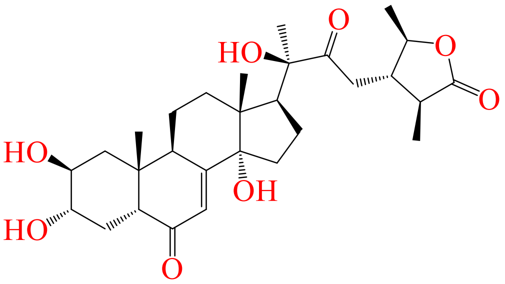
271.	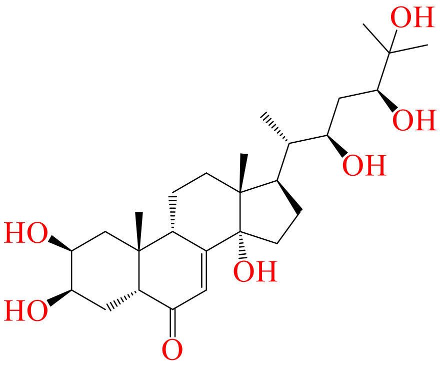
272.	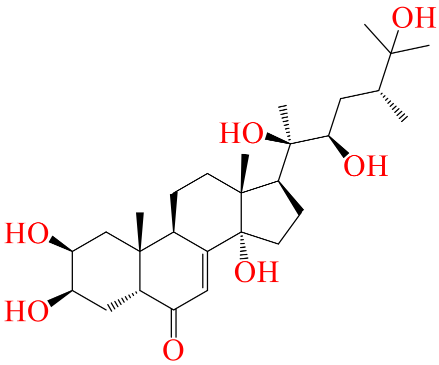
273.	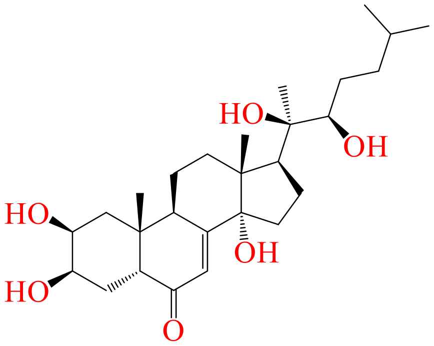

274.	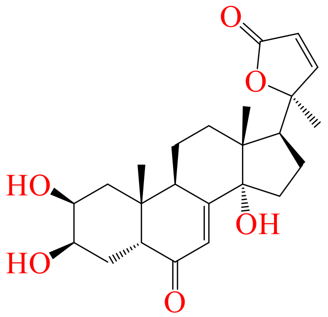
275.	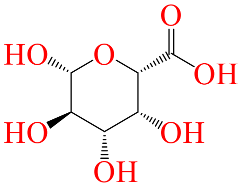
276.	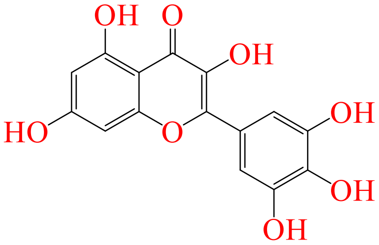
277.	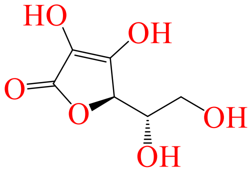
278.	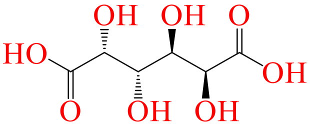
279.	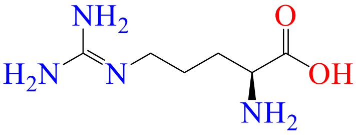
280.	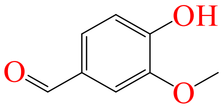
281.	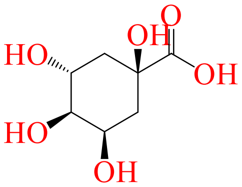
282.	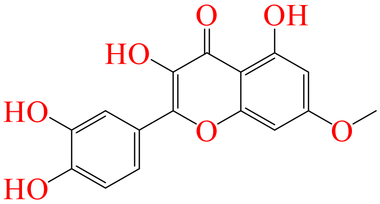

283.	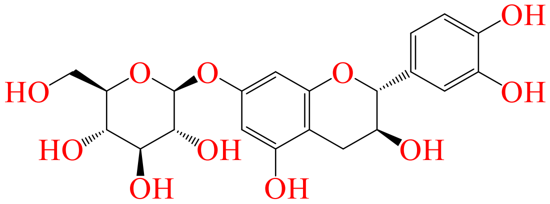
284.	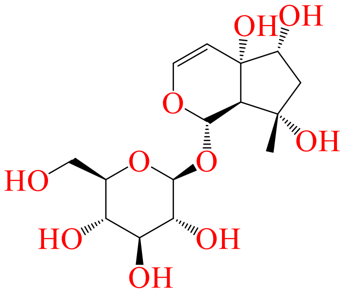
285.	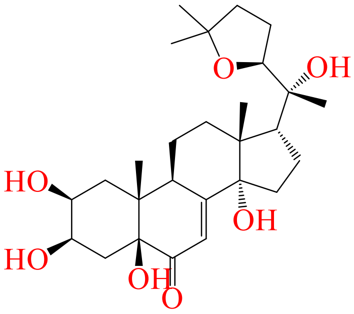
286.	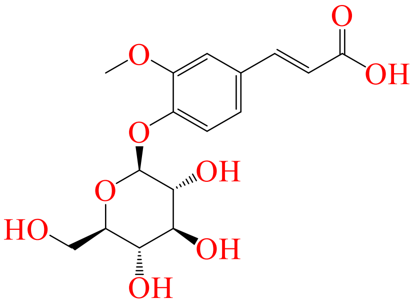
287.	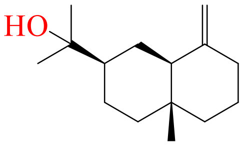
288.	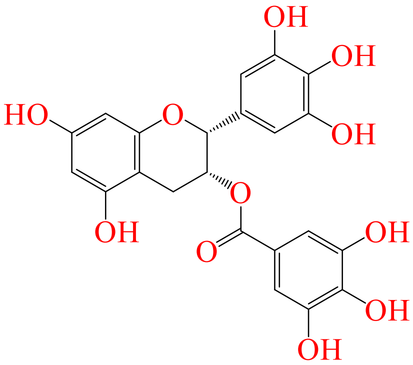

289.	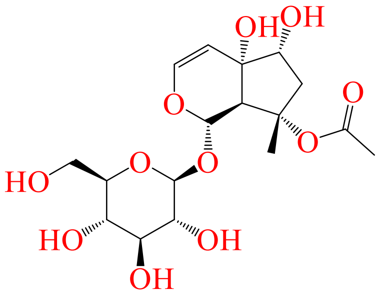
290.	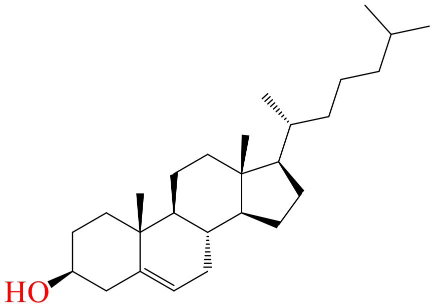
291.	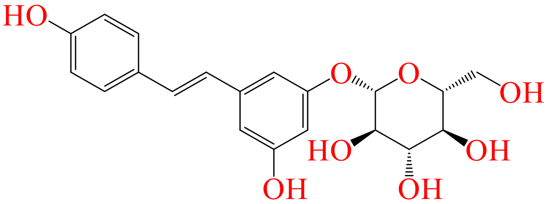
292.	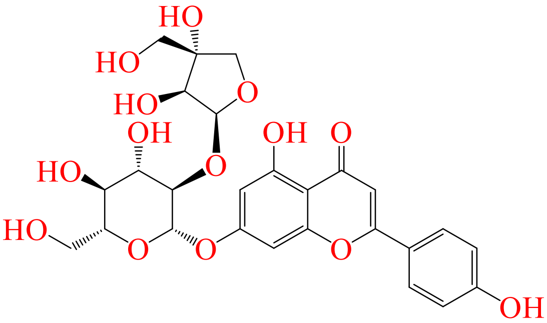
293.	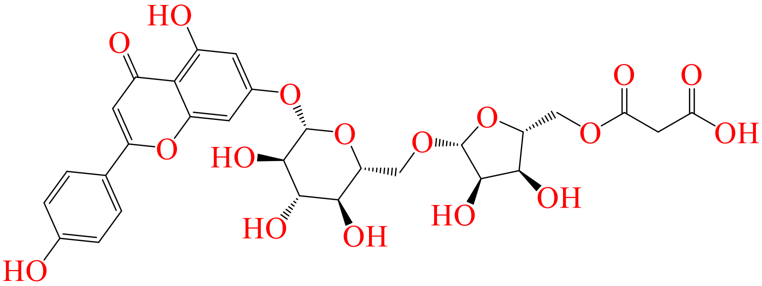
294.	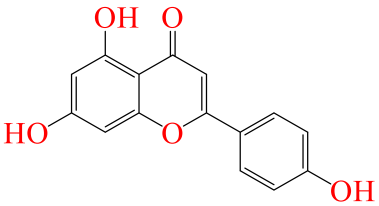

295.	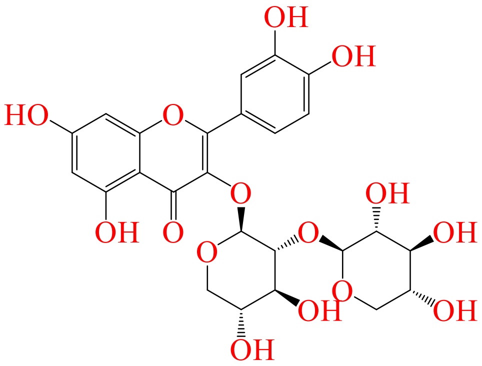
296.	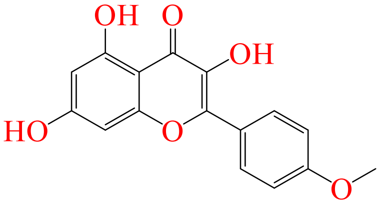
297.	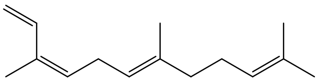
298.	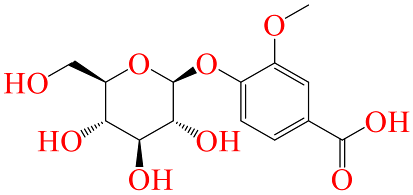
299.	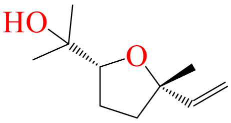
300.	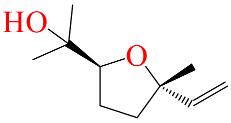
301.	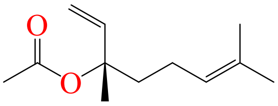
302.	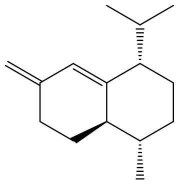
303.	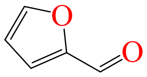
304.	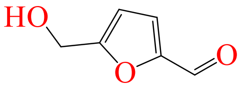
305.	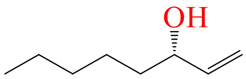
306.	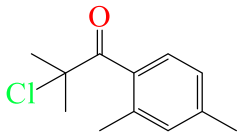

307.	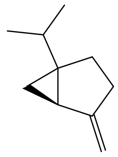
308.	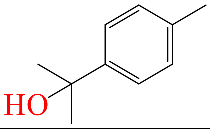
309.	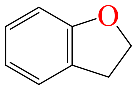
310.	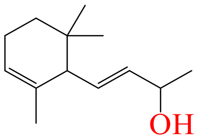
311.	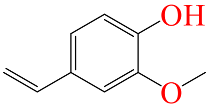
312.	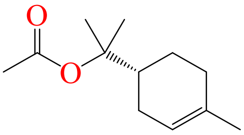
313.	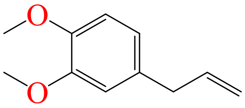
314.	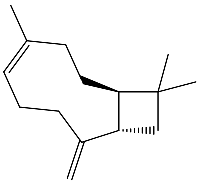
315.	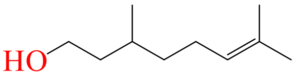
316.	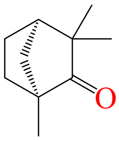
317.	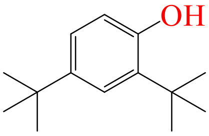
318.	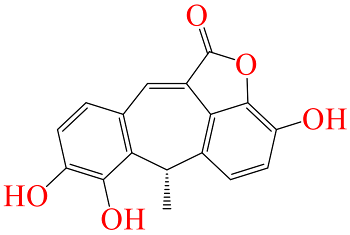

319.	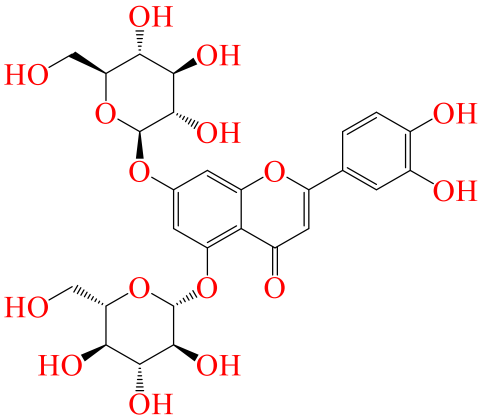
320.	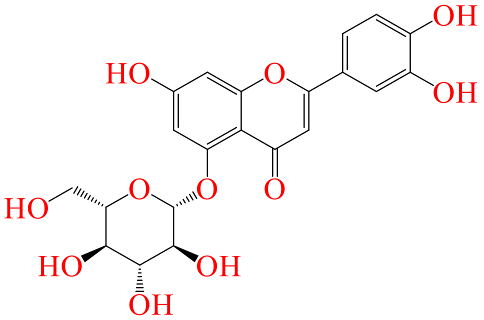
321.	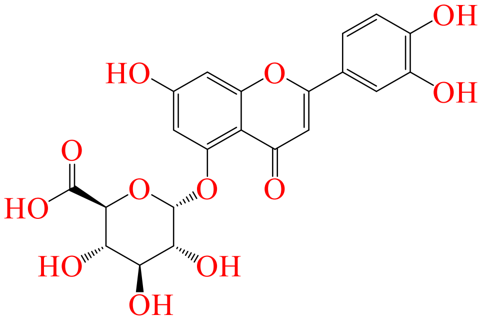
322.	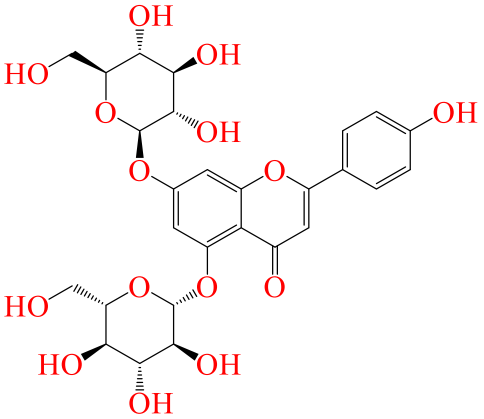
323.	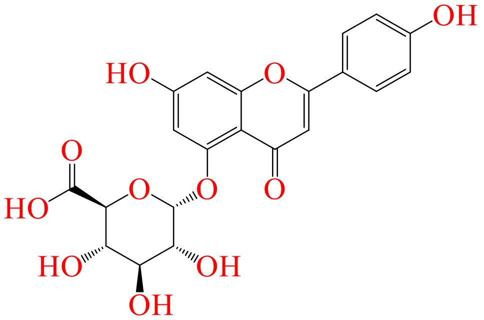

324.	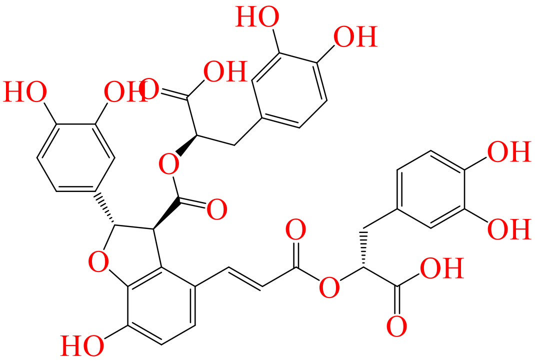
325.	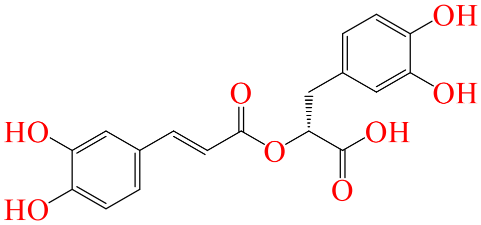
326.	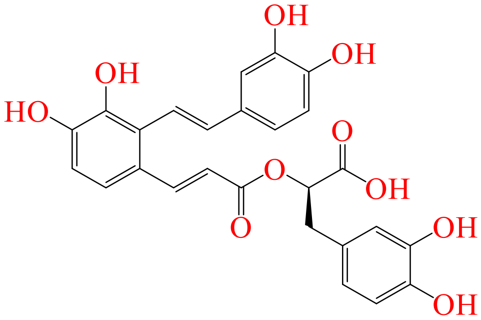
327.	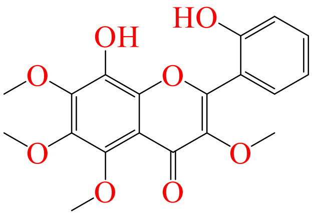
328.	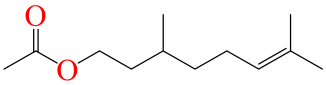
329.	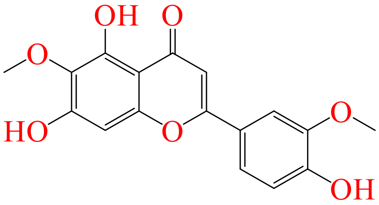
330.	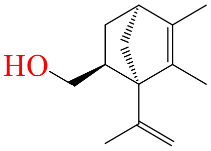
331.	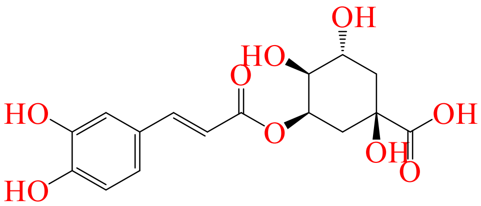

332.	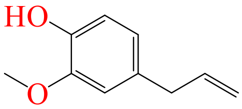
333.	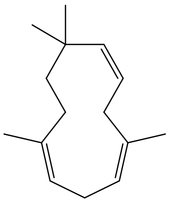
334.	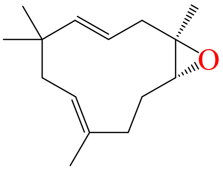
335.	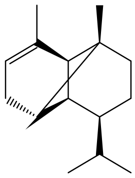
336.	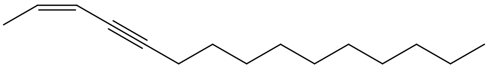
337.	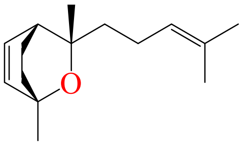
338.	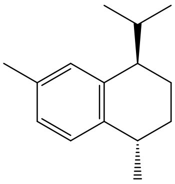
339.	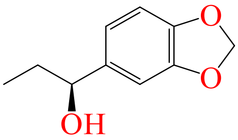
340.	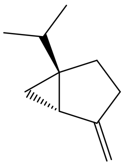
341.	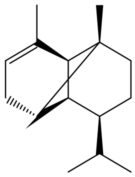
342.	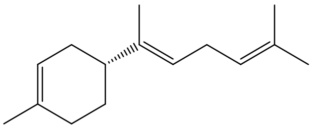

343.	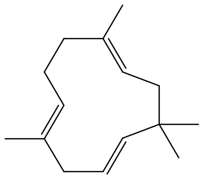
344.	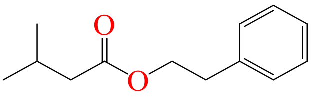
345.	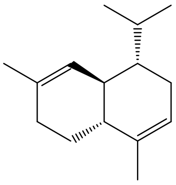
346.	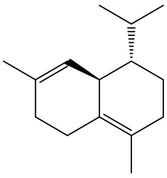
347.	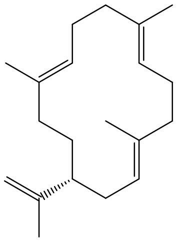
348.	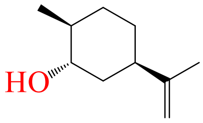
349.	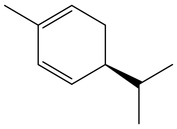
350.	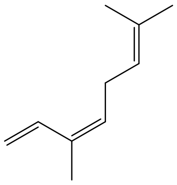
351.	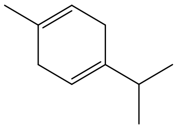
352.	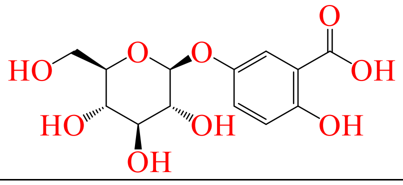
353.	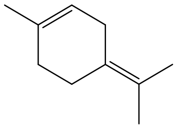
354.	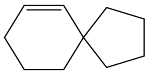

355.	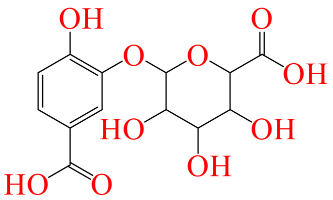
356.	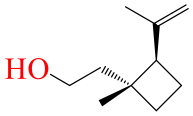
357.	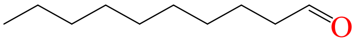
358.	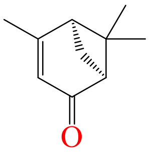
359.	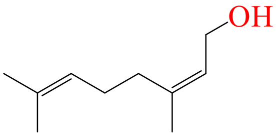
360.	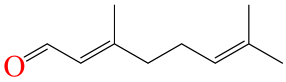
361.	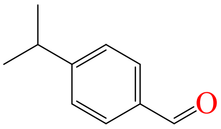
362.	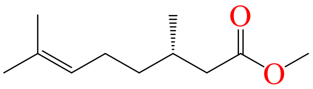
363.	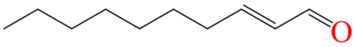
364.	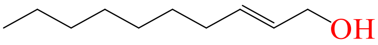
365.	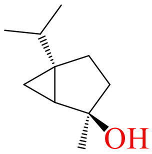
366.	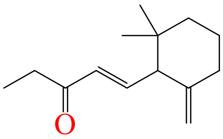
367.	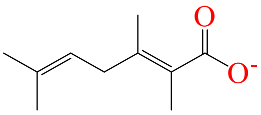
368.	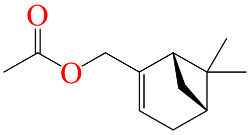
369.	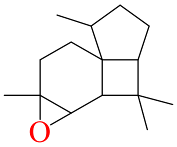

370.	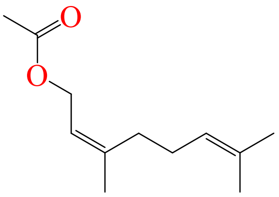
371.	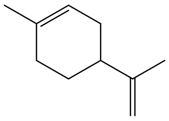
372.	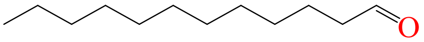
373.	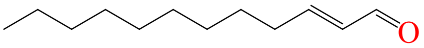
374.	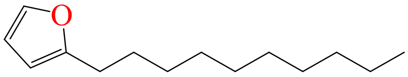
375.	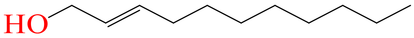
376.	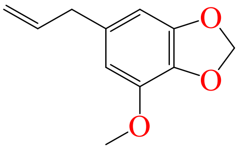
377.	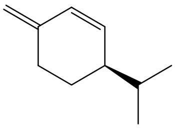
378.	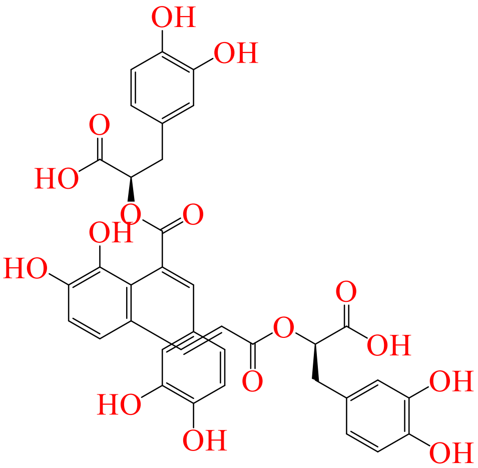
379.	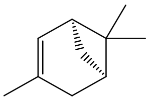
380.	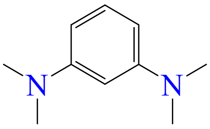
381.	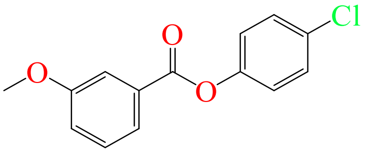
382.	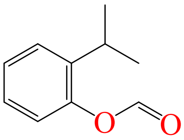

383.	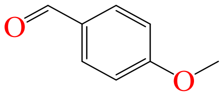
384.	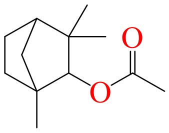
385.	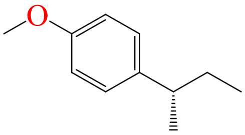
386.	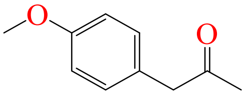

References

[1] A. Berrani *et al.*, 'Anabasis aretioides Coss. & Moq. phenolic compounds exhibit in vitro hypoglycemic, antioxidant and antipathogenic properties', *Journal of Basic and Clinical Physiology and Pharmacology*, vol. 30, no. 2, pp. 251–257, Mar. 2019, doi: 10.1515/jbcpp-2018-0154.

[2] S. Haida, A. Kribii, and A. Kribii, 'Chemical composition, phenolic content and antioxidant capacity of *Haloxylon scoparium* extracts', *South African Journal of Botany*, vol. 131, pp. 151–160, Jul. 2020, doi: 10.1016/j.sajb.2020.01.037.

[3] F. Z. Jawhari *et al.*, 'Anacyclus pyrethrum (L): Chemical Composition, Analgesic, Anti-Inflammatory, and Wound Healing Properties', *Molecules*, vol. 25, no. 22, Art. no. 22, Jan. 2020, doi: 10.3390/molecules25225469.

[4] A. Baslam *et al.*, 'Phytochemistry, Antioxidant Potential, and Antibacterial Activities of Anacyclus pyrethrum: Promising Bioactive Compounds', *Horticulturae*, vol. 9, no. 11, Art. no. 11, Nov. 2023, doi: 10.3390/horticulturae9111196.

[5] K. E. Mokhtari, M. E. Kouali, M. Talbi, L. Hajji, and A. E. Brouzi, 'Chemical composition and insecticidal activity of Anacyclus pyrethrum essential oil from the Bensliman area against Culex pipiens', *Mediterranean Journal of Chemistry*, vol. 10, no. 1, Art. no. 1, Jan. 2020, doi: 10.13171/mjc10102021l198kem.

[6] F. Z. Jawhari *et al.*, 'Phytochemical, Morphological and Genetic Characterisation of Anacyclus pyrethrum var. depressus (Ball.) Maire and Anacyclus pyrethrum var. pyrethrum (L.) Link', *Molecules*, vol. 28, no. 14, Art. no. 14, Jan. 2023, doi: 10.3390/molecules28145378.

[7] S. Amine *et al.*, 'Influence of Abiotic Factors on the Phytochemical Profile of Two Species of Artemisia: A. herba alba Asso and A. mesatlantica Maire', *International Journal of Plant Biology*, vol. 13, no. 2, Art. no. 2, Jun. 2022, doi: 10.3390/ijpb13020007.

[8] I. Sbai *et al.*, 'Chemical composition and antioxidant activity of the essential oil of Artemisia vulgaris from Morocco', Nov. 2019.

[9] H. El Hajjouji, E. Pinelli, M. Guiresse, G. Merlina, J.-C. Revel, and M. Hafidi, 'Assessment of the genotoxicity of olive mill waste water (OMWW) with the *Vicia faba* micronucleus test', *Mutation Research/Genetic Toxicology and Environmental Mutagenesis*, vol. 634, no. 1, pp. 25–31, Dec. 2007, doi: 10.1016/j.mrgentox.2007.05.015.

[10] A. Bouyahya *et al.*, 'Ethnomedicinal use, phytochemistry, pharmacology, and toxicology of *Ajuga iva* (L.,) schreb', *Journal of Ethnopharmacology*, vol. 258, p. 112875, Aug. 2020, doi: 10.1016/j.jep.2020.112875.

[11] S. El Kharraf, M. L. Faleiro, F. Abdellah, S. El-Guendouz, E. M. El Hadrami, and M. G. Miguel, 'Simultaneous Hydrodistillation-Steam Distillation of Rosmarinus officinalis, Lavandula angustifolia and Citrus aurantium from Morocco, Major Terpenes: Impact on Biological Activities', *Molecules*, vol. 26, no. 18, Art. no. 18, Jan. 2021, doi: 10.3390/molecules26185452.

[12] B. Soulaimani *et al.*, 'Chemical composition, antimicrobial activity and synergistic potential of essential oil from endemic *Lavandula maroccana* (Mill.)', *South African Journal of Botany*, vol. 125, pp. 202–206, Sep. 2019, doi: 10.1016/j.sajb.2019.07.030.

[13] A. Nafis *et al.*, 'Chemical composition and synergistic effect of three Moroccan lavender EOs with ciprofloxacin against foodborne bacteria: a promising approach to modulate antimicrobial resistance', *Letters Applied Microbiology*, vol. 72, no. 6, pp. 698–705, Jun. 2021, doi: 10.1111/lam.13460.

[14] A. Bouymajane *et al.*, 'Phenolic Compounds, Antioxidant and Antibacterial Activities of Extracts from Aerial Parts of Thymus zygis subsp. gracilis, Mentha suaveolens and Sideritis incana from Morocco', *Chemistry & Biodiversity*, vol. 19, no. 3, p. e202101018, 2022, doi: 10.1002/cbdv.202101018.

[15] J. El-Akhal, A. P. Oliveira, R. Bencheikh, P. Valentão, P. B. Andrade, and M. Morato, ‘Vasorelaxant Mechanism of Herbal Extracts from Mentha suaveolens, Conyza canadensis, Teucrium polium and Salvia verbenaca in the Aorta of Wistar Rats’, *Molecules*, vol. 27, no. 24, Art. no. 24, Jan. 2022, doi: 10.3390/molecules27248752.
[16] A. Ainane *et al.*, ‘Chemical composition and insecticidal activity of five essential oils: Cedrus atlantica, Citrus limonum, Rosmarinus officinalis, Syzygium aromaticum and Eucalyptus globules’, *Materials Today: Proceedings*, vol. 13, pp. 474–485, Jan. 2019, doi: 10.1016/j.matpr.2019.04.004.
[17] ‘The Needles of Aleppo Pine From the Province of … — Library of Science’. Accessed: Mar. 01, 2024. [Online]. Available: https://bibliotekanauki.pl/articles/2202321
[18] K. A. Amrani, M. Barbouchi, M. Elidrissi, A. Amechrouq, and M. Chokrad, ‘Chemical composition and physicochemical properties of the essential oil of coriander (Coriandrum sativum L.) grown in Morocco’, *RHAZES: Green and Applied Chemistry*, vol. 4, no. 4, Art. no. 4, May 2019, doi: 10.48419/IMIST.PRSM/rhazes-v4.16202.
[19] M. Barrahi *et al.*, ‘Chemical composition and evaluation of antibacterial activity of fennel (Foeniculum vulgare Mill) seed essential oil against some pathogenic bacterial strains’, *Caspian Journal of Environmental Sciences*, vol. 18, no. 4, pp. 295–307, Oct. 2020, doi: 10.22124/cjes.2020.4276.
[20] M. Abdellaoui, E. dine T. Bouhlali, M. Derouich, and L. El-Rhaffari, ‘Essential oil and chemical composition of wild and cultivated fennel (*Foeniculum vulgare* Mill.): A comparative study’, *South African Journal of Botany*, vol. 135, pp. 93–100, Dec. 2020, doi: 10.1016/j.sajb.2020.09.004.
[21] A. A. Maofari *et al.*, ‘CHEMICAL COMPOSITION AND ANTIBACTERIAL PROPERTIES OF ESSENTIAL OILS OF Pimpinella Anisum L. GROWING IN MOROCCO AND YEMEN’, 2013.
[22] Y. Moustakime, Z. Hazzoumi, and K. Amrani Joutei, ‘Aromatization of virgin olive oil by seeds of *Pimpinella anisum* using three different methods: Physico-chemical change and thermal stability of flavored oils’, *Grain & Oil Science and Technology*, vol. 4, no. 3, pp. 108–124, Sep. 2021, doi: 10.1016/j.gaost.2021.07.001.
[23] M. Derouich, E. D. T. Bouhlali, M. Bammou, A. Hmidani, K. Sellam, and C. Alem, ‘Bioactive Compounds and Antioxidant, Antiperoxidative, and Antihemolytic Properties Investigation of Three *Apiaceae* Species Grown in the Southeast of Morocco’, *Scientifica*, vol. 2020, pp. 1–10, Sep. 2020, doi: 10.1155/2020/3971041.
[24] A. E. Mostaphi, H. E. Hartiti, M. Barrahi, A. Zarrouk, M. Berrabeh, and M. Ouhssine, ‘Etude Physico-chimiques et Analyses Chromatographiques de l’huile Essentiel des Grains de céleri (Apium Graveolens.L) [Physico-chemical and Chromatographic Analysis Study of the Essential oil of Celery Seed (Apium Graveolens.L)]’.
[25] I. Es-safi *et al.*, ‘An Insight into the Anxiolytic and Antidepressant-Like Proprieties of Carum carvi L. and Their Association with Its Antioxidant Activity’, *Life*, vol. 11, no. 3, Art. no. 3, Mar. 2021, doi: 10.3390/life11030207.
[26] J. Kouar *et al.*, ‘Comparison between electrocoagulation and solvent extraction method in the process of the dechlorophyllation of alcoholic extracts from Moroccan medicinal plants Petroselinum crispum, Thymus satureioides and microalgae Spirulina platensis’, *SN Appl. Sci.*, vol. 1, no. 1, p. 132, Jan. 2019, doi: 10.1007/s42452-018-0137-1.
